# Does financial literacy matter for firms? An empirical investigation on Sri Lankan SMEs

**DOI:** 10.1371/journal.pone.0354031

**Published:** 2026-07-21

**Authors:** Geesara Kasthuri Arachchi, Ishan Perera, Chathurya Gamage, Shehara Wijesinghe, Krishantha Wisenthige, Nirmani Dayapathirana

**Affiliations:** Sri Lanka Institute of Information Technology, Malabe, Sri Lanka; Universiti Teknologi MARA, MALAYSIA

## Abstract

Small and Medium Enterprises (SMEs) are vital to economic growth and job creation, especially in developing countries. However, their potential is not fully utilized due to challenges related to limited financial inclusion, poor resource management, and underdeveloped capital markets. Though financial literacy is well-researched in personal finance, its role as a knowledge resource at the firm level and its impact on firm performance remain underexplored. This study investigates the influence of owner-managers’ financial literacy on firm performance, focusing on the mediating roles of FinTech adoption and access to finance. By taking a sample of 264 SME owner-managers in the Western Province of Sri Lanka, this study found that financial literacy significantly enhances firm performance (*β* = 0.241, *p* < .01). The findings indicate that financial literacy supports sound financial decision-making and risk mitigation and is positively associated with both FinTech adoption (*β* = 0.542, *p* < .001) and access to finance (*β* = 0.533, *p* < .001). These results underscore the necessity of enhancing financial literacy and promoting FinTech adoption to fully realize the potential of SMEs within developing economies. The study contributes a concise yet impactful framework for enhancing the performance of SMEs through the strategic application of financial literacy. Based on the study’s findings, it is recommended that enterprise owners and managers prioritize achieving an adequate level of financial literacy. Furthermore, governmental institutions should revise their financial literacy programs, reconsidering their design and implementation strategies to effectively engage key personnel within enterprises. Current programs appear underutilized due to deficiencies in depth and practical applicability of the knowledge given in them.

## Introduction

Given that the small and medium-sized enterprises (SMEs) constitute the backbone of most global economies, a robust SME sector is indispensable for sustained and steady economic growth [1–4]. Thus, the determination of factors influencing firm growth and firm performance is imperative to optimize their potential to empower global economies [3]. Firm characteristics, firm strategy, public support, and macroeconomic environmental factors are the most influential factors affecting SMEs, and these factors can interplay differently in different contexts [5]. Sri Lankan SMEs face several specific challenges, including limited access to formal financial services due to stringent collateral requirements, high interest rates, and bureaucratic hurdles, which hinder their ability to secure necessary funding [6–8]. Additionally, socio-cultural factors such as reliance on family support and influence from religious leaders significantly impact entrepreneurial outcomes in the country [6]. Beyond the said challenges, Sri Lankan SMEs are substantially impacted by elevated inflation rates [9]. This inflationary pressure leads to increased operational costs across various facets of their businesses, thereby compromising their economic viability and long-term sustainability. While external factors and external challenges are beyond a firm’s control, internal factors such as managerial capabilities are within its influence to resist these challenges.

The capabilities of managers play a crucial role in shaping a firm’s direction, as they are the ones who employ business practices from top to bottom in the firm [10]. Managerial capabilities of managers play a vital role in shaping a firm’s strategy, innovation, and overall performance. Firms need to constantly adapt and evolve to survive and thrive [11]. These dynamic capabilities include sensing and seizing opportunities, and transforming the organization to capitalize on it. In the skillset of a manager, this study suggests that financial literacy should be a foundational skill. From a global perspective, only 39% of adults are able to score a minimum of financial literacy, indicating that overall financial literacy isn’t that great either [12]. Especially in developing countries, Turkey, Mexico, Colombia, Lebanon, and Uruguay exhibit the lowest financial literacy [13]. In the Sri Lankan context, only 57.9% of the population is financially literate [14]. Regardless of the mediocre financial literacy score, it is reported that Sri Lankan entrepreneurs and investors have poor financial literacy [15,16]. Hence, the likelihood of negative effects of low financial literacy is higher than ever.

Financial literacy has been linked to better personal wealth management and overall improvement in financial decision-making on an individual-level [17–20]. Financial literacy has been linked to superior financial well-being as well [20]. Despite the dominant literature on financial literacy and personal finance, there is a lack of studies on financial literacy at the firm level and its contribution to the organization as a knowledge resource. However, in the last few years, scholarly attention has surged in the SME research domain. Empirical investigations have suggested financial literacy linkage to technological innovations, growth, performance, and technological adaptations of firms [21–24]. Yet, what remains underexplored is how financial literacy at the owner-manager level specifically influences firm performance through modern financial tools such as FinTech and improved access to finance, especially in the context of developing economies. Prior research either isolates these factors or focuses on developed markets, leaving a void in understanding their combined effect in developing economies like Sri Lanka.

Studies suggest that owner-managers’ financial illiteracy demonstrated a lack of attention to the financing of a firm [25]. Not prioritizing optimal financing for a firm is a grave error that the management of a firm should strive to avoid, as financing has a lasting impact on the performance of a firm [26]. Financial constraints pose a particularly formidable barrier to firm growth within developing countries, which limits the growth and performance of SMEs [27–29]. Lack of, or if not inability to meet, short-term and long-term debt is one of the key drivers of SME failure [30]. Adequate financial resources empower SMEs to take strategic initiatives such as business expansion, technology adoption, innovations, and R&D [31,32]. Due to undeveloped capital markets in developing countries, SMEs find difficulty in financing their business at reasonable prices [33]. Following the COVID-19 pandemic, central banks have implemented interest-rate hikes to combat inflationary pressures [34]. This has resulted in increased borrowing costs for businesses due to higher interest rates on loans, and lenders have become more risk-averse, leading to tighter credit conditions and further escalating borrowing costs, particularly for SMEs [34]. Therefore, optimal financial decision-making has become more critical than ever, as financial markets are increasingly unforgiving. Most Asian SMEs struggle to participate in capital markets due to information asymmetry [35]. Hussain, Salia [36] argued that financial literacy strongly contributes to resolving the information asymmetry between lenders and borrowers. Financial literacy has been a promising element in elevating financial exclusion to SMEs as it has been linked to relieving financial constraints for SMEs in both developing and developed economies [1,36–38].

On top of this, firms do not necessarily need to rely on conventional banking. Financial technology (FinTech), defined as the use of digital technologies to deliver financial services such as payments, lending, and financing in a more efficient and accessible manner, offers alternative solutions to traditional financial services, including peer-to-peer lending and mobile money, thereby transforming the business landscape [39]. Traditional banking services fall short due to heavy regulations and long processes when assessing a borrower. FinTech lenders, on the other hand, can analyze borrowers’ information more efficiently and offer loans to people who might not qualify for traditional bank loans [40]. FinTech reduces shortcomings of traditional financial services, such as assessing creditworthiness, and increases the performance of firms [39]. The question arises in this case, the composition of financial awareness by the financially literate managers, are they more likely to adopt technologies like FinTech into their firm? Studies suggest that those financially literate embody increased awareness of FinTech products [41–43], while some demonstrate that financial literacy does not have a direct impact on FinTech adoptions [44]. Building upon that knowledge, this study aims to investigate FinTech adoption and the financial literacy relationship at a firm level. This is another underexplored area that has limited knowledge in current literature. Addressing this gap is crucial for policymakers and SME stakeholders who seek to design effective financial education programs and promote strategic FinTech adoption to strengthen firm performance.

The purpose of this study is to assess the impact of owner-manager financial literacy on access to finance and FinTech adoption in shaping firm performance. As demonstrated by Goyal and Kumar [45], so far a limited number of studies have evaluated these factors in combination with financial literacy in developing economies against studies in developed economies, and even extremely low counts of studies in Sri Lanka. Examining the influence of owner-managers’ financial literacy on firm performance within the Sri Lankan context is valuable due to the country’s well-documented economic challenges and instability. Understanding how financial literacy enables owner-managers to navigate external economic threats is essential for enhancing firm resilience and sustainability in such an environment. The relationship between financial literacy and FinTech adoption is inconclusive and mixed. Scholars have revealed that this relationship is weak, and suggests basic understanding of financial literacy may not translate to meaningful effects on FinTech adoption [43,44]. This study argues that this is due to financial literacy scale disparities; traditional and basic financial literacy scales do not capture the financial literacy required in a business environment. In addition, financial literacy on specific FinTech products and services that offer alternative financing options is scarce. In light of the above, this study aims to create a framework for relieving financial constraints through stimulating financial literacy for SMEs.

This study makes several contributions by providing insights into the role of financial literacy as a knowledge resource at the firm level. Firstly, this study examines the direct relationship between owner-manager financial literacy and FinTech adoption, as this relationship is inconclusive in literature. Secondly, this study investigates the owner-manager financial literacy relationship in accessing funds for the firm, complementing the findings of previous studies. Thirdly, this study investigates the direct relationship between financial literacy and firm performance as well as the mediating roles of FinTech adoption and access to finance in the said relationship, which has limited knowledge in literature. Finally, this study employs a financial literacy scale to measure the financial literacy required for effective financial management within a firm, while also reflecting the organizational context. This approach provides a precise assessment of financial literacy in firm settings and improves comparability across studies.

The paper is structured as follows, [href:#_Literature_review,_theory,]section 2 provides the current knowledge in the literature and the theoretical grounds for the hypotheses, [href:#_Methods]section 3 contains methodological approaches used in the study, [href:#_Results]section 4 contains the results, [href:#_Discussion]section 5 contains the discussions related to the findings, and lastly [href:#_Conclusion]section 6 contains the concluding statements, implications, limitations and future research.

### Literature review, theory, and research hypotheses

The literature review was conducted through a systematic approach to identify relevant studies and theoretical frameworks that explore the relationship between financial literacy, access to finance, FinTech adoption, and SME performance. Journal articles were carefully analyzed to understand the existing research gaps and key findings. This review guided the development of the theoretical model and research hypotheses by integrating insights from prior studies and aligning these with the context of SMEs in developing economies. The focus was to establish a robust foundation that links financial literacy to firm performance while considering the mediating roles of access to finance and FinTech adoption.

### Financial literacy and firm performance

Operationalization of financial literacy is an arduous task; many scholars have different interpretations and definitions. The Organization for Economic Cooperation and Development (OECD [12] defines financial literacy as having the awareness, knowledge, skills, attitudes, and behaviors to make smart financial choices. Lusardi [46], Lusardi and Mitchell [47] define financial literacy as “people’s ability to process economic information and make informed decisions about financial planning, wealth accumulation, pensions, and debt”. Financial literacy has been linked to better personal wealth management, such as improved stock-market participation, which exerts effects on savings behavior and borrowing behavior practices [17–19,48,49]. For instance, Lusardi and Tufano [50] calculated that the least financially literate in thein the United States of America (USA)(29% of cardholders) account for 42% of credit card charges, paying 50% higher fees than the average cardholder. One-third of these fees are directly attributable to low financial literacy. Financially literate individuals are about twice as likely to pay below-average interest rates when borrowing, whether for credit cards or mortgages [51]. Low financial literacy has also been linked to suboptimal financial behavior, such as inadequate retirement planning and carrying debt all the way to retirement [46,52]. Contributing to that point, the financially illiterate tend to obtain higher interest-rate loans [50]. Thus, inadequate financial literacy precipitates detrimental financial outcomes, thereby directly impinging upon an individual’s financial well-being.

While a substantial body of research exists on the relationship between individual financial literacy and personal finance management [17,48,49], scant attention has been given to understanding the extent to which these competencies translate to effective financial decision-making within the context of businesses’ financial management. The prevalent theoretical explanation for the link between financial literacy and firm performance and related constructs centers on a resource-based view theory [22]. This study utilizes the Knowledge-Based View (KBV) theory. The KBV can be identified as the most appropriate theoretical framework for examining the relationship between financial literacy and firm performance or firm growth, as it conceptualizes knowledge as the most strategically important resource. According to the KBV, the ability to create, integrate, and apply knowledge is critical in gaining and sustaining competitive advantage [53,54] and aligning knowledge resources with strategic requirements is paramount [54]. The theory emphasizes two types of knowledge: tacit knowledge (hard to articulate, experience-based knowledge) and idiosyncratic knowledge (unique knowledge). Furthermore, those two types of knowledge cause causal ambiguity and path dependency, which are likely to be irreproducible by an external party such as a competitor.

Drawing from these theoretical grounds, financial literacy has been framed as a strategic knowledge resource that demonstrates primary characteristics of the KBV. Financial Literacy incorporates experience-based knowledge and specialized knowledge regarding financial markets, how to allocate resources efficiently, assess investment performance, and to manage risk in the area/domain of finance. Financial Literacy is primarily tacit in nature, which means that it cannot be fully transferred or duplicated by formal education alone; rather, it is developed through experience and context-based application. Therefore, financially literate owner-managers develop knowledge-based assets that are hard for others to replicate or imitate, thus providing firms with potential sources of competitive advantage.

Financial literacy empowers individuals and organizations by providing the necessary knowledge base for informed financial decision-making, effective resource management, and the alignment of financial strategies with overall business objectives. Financial literacy can be identified as a knowledge element that can be utilized to improve financial behavior and financial decision-making. Drexler, Fischer [55] found that micro-entrepreneurs who received simplified rules of thumb training showed significant improvements in managing their financing. Participants who received the training demonstrated a hike in salesduring periods of economic downturn for the firm. Furthermore, they exhibited a greater likelihood of maintaining financial records, a crucial aspect for assessing the overall financial health of their businesses. Financial literacy is crucial for entrepreneurs to effectively manage daily operations and gain a competitive advantage [56]. Widdowson and Hailwood [57] state that financial literacy is particularly useful for smaller firms and benefits from budgeting, investment, and borrowing knowledge that comes with financial literacy. Financial literacy contributes to a firm’s improved access to financing and enhanced risk management capabilities [58]. This subject will be further elaborated upon in the subsequent section.

Works by Eniola, Entebang [59] and Eniola and Entebang [60] posited that financially literate owner-managers contribute largely to improved firm performance. They argued that this impact stems from the ability of financially literate entrepreneurs to make informed decisions regarding resource procurement, allocation, and utilization to develop and implement effective financial strategies. Diéguez-Soto, Martínez-Romero [61] add to the argument that the presence of financial knowledge enhances financial behavior and therefore leads to a rise in the pursuit of investment opportunities, leading to better firm growth. Financial literacy helps identify financial aspects of business decisions and enables SMEs to cope with economic transitions [62]. Agyapong and Attram [22] state that improved financial literacy empowers individuals to make sound financial decisions, optimize resource allocation, reduce waste, and effectively manage business risks, ultimately fostering a competitive advantage and stronger firm growth. The financially literate are suggested to have a broader and deeper understanding of financial opportunities and risks, influencing their strategic orientations towards innovation, resource allocation, and risk-taking [23].

Financial knowledge is undeniably crucial, but the financial attitude that comes with financial literacy toward financing, risk-taking, and business challenges is important in shaping business outcomes [60]. However, the positive impacts of financial literacy extend beyond merely improving financial outcomes. Financial literacy has been surprisingly linked with better innovation capability for firms [63,64]. Financial literacy has been linked to successful product development and sustainable competitive performance, stating that financially literate managers are better equipped to make sound judgments regarding financial viability when developing new products and resource allocations [65]. Specifically, increased financial literacy helps SMEs in setting clear objectives, evaluating market trends, and formulating contingency plans, key components of effective planning [66]. Therefore, financially literate managers are better equipped to conduct risk assessments and informed cost assessments regarding firms’ strategic decisions [66].

While financial literacy can be a valuable tool, the presence of financial literacy may not translate into firm growth in all contexts. Its impact can vary across different contexts, and other factors may play a more significant role, especially in developing and underdeveloped economies, as other factors could diminish the importance of financial literacy since macroeconomic factors can drive firm performance [5,60]. The KBV posits that firm performance is driven by the effective creation, integration, and application of knowledge-based resources. Consistent with KBV, the value of knowledge is context-dependent and does not generate strategic advantage in isolation. In the present study, financial literacy is conceptualized as a firm-level knowledge resource that enhances decision-making and operational effectiveness, rather than as an individual-level attribute. Accordingly, the contribution of financial literacy to business outcomes is contingent upon how such knowledge is embedded and utilized within organizational processes. Aligning with KBV and prior empirical evidence, this study proposes the following hypothesis.

H_1_: Financial literacy has a positive impact on firm performance

### Financial literacy, access to finance, and firm performance

Among numerous hurdles that limit firm growth and performance, financial constraints are a major hurdle [29]. External financing is a catalyst for innovation [67–69], and financial deepening can encourage firm incorporation, unlocking benefits like risk diversification and limited liability [70]. SMEs are more likely to be credit-constrained than larger firms [71]. Financial access is especially critical for younger SMEs [72]. Smaller firms inherently possess less capacity to absorb risks, meaning that even minor financial setbacks can have severe, potentially catastrophic consequences, disrupting the performance of the firm. This contrasts sharply with larger corporations, which typically have greater financial buffers and diversified operations that allow them to withstand or recover from adverse events more readily.

One of the key issues for firms when accessing external finance is information asymmetry, which is also the foundation of the pecking order theory [73]. Investors and lenders rely heavily on their assessment of a firm’s value before committing capital. A fundamental assumption underlying this process is that the firm’s management possesses a more comprehensive understanding of the firm’s true worth compared to external parties [73]. Information asymmetry could affect the supply-side during financial transactions, where lenders possess less insight into the borrowers’ repayment capability and details of their businesses’ financial health. This may lead to financial institutions perceiving SMEs as risky choices for lending funds, tightening lending terms, and elevating interest rates. SMEs could often face supply-side constraints due to high interest rates and collateral requirements [74]. Lack of financial literacy leads to poor financial transparency, posing a challenge for lending institutions in assessing creditworthiness, consequently marginalizing SMEs from formal finance [75–77]. Furthermore, MSMEs are specifically struggling to maintain financial transparency as they are less likely to keep financial records, which contributes to poor financial communication [77–80]. Financial literacy can be attributed as a step forward, especially in reducing the information asymmetry between borrowers and lenders [36]. Financially literate individuals are more likely to understand debt information, conduct debt reporting [81], and more likely to keep financial records [55]. Thus, financial literacy can be considered as one of the factors that could alleviate information asymmetry, stimulating better financial access for a firm. Financially literate firms seem to be better able to negotiate favorable loan terms and are less often subjected to restrictions such as higher interest rates (price rationing) or being given less money than they applied for (amount rationing) [82]. For instance, access to credit in Kerala is hampered not by a lack of available products, but by a dual problem: insufficient financial literacy among owners and often unfair credit evaluation [83].

Individuals with higher financial literacy are better equipped to comprehend financial information pertaining to debt products and services. This enhanced understanding is hypothesized to spur increased utilization of these financial products and services, while simultaneously facilitating more effective communication of the firm’s value to external funding sources. Supporting that argument, financial literacy programs have been seen to increase financial inclusion [84]. Individuals who possess financial literacy are seen to be less likely to be excluded financially [85,86]. For SMEs especially, a sophisticated understanding of the financial consequences of financial decisions is not merely beneficial but imperative for sustained success, as they possess less capacity to resist financial disasters. This advanced financial knowledge comes with financial literacy underpins informed decision-making, acts as a crucial preventative measure against catastrophic financial decisions promotes both enduring growth and organizational resilience [21]. Additionally, Singh [87] has found that financial literacy is linked to the usage of finances as well. The author identified that many rural individuals are unaware of proper utilization of credit services, often resulting in poor decision-making and reluctance to borrow from formal institutions. Similarly, financial literacy is suggested to improve access to finance indirectly through the utilization of financial literacy [38], interpreting that financial literacy may possess confidence in their ability to navigate financial systems and trust in the financial systems. Notably, financial knowledge does not appear to reduce individuals’ reluctance to take risks in financial matters. Instead, those with higher financial understanding tend to be more thoughtful and well-informed when considering uncertain financial options [88]. This suggests that enhanced financial literacy adopts a more calculated approach to risk, rather than a diminished aversion to it, which is crucial because the risk-taking landscape for SMEs is significantly less forgiving.

Kodongo [89] finds that financial literacy has also been associated with improved access to formal financing for individuals. However, Candiya Bongomin, Munene [90] reveal that not all components of financial literacy correlate with financial inclusion, but only the financial attitude. Thus, financial literacy may foster a propensity for individuals to actively seek out financial opportunities, thereby increasing their engagement within financial markets. Furthermore, enhanced financial literacy may facilitate more informed and discerning evaluations of available financial options. Susan [91] states that even though financial literacy indirectly affects the growth of a firm through financial access, there can be nuances to this relationship, such as the economic conditions, quality, and affordability of the financial services and products. Synthesizing these observations, financial literacy is crucial in alleviating information asymmetry for firms. Furthermore, it appears to facilitate the actual utilization of financial services, thereby fostering enhanced awareness critical for making informed financing decisions. This heightened understanding enables individuals and entities to approach risky financial options with a more informed perspective. Consistent with prior scholarly investigations, the following hypothesis is proposed.

H_2_: Financial literacy has a positive impact on access to finance

H_3_: Access to finance positively impacts firm performance

H_4_: Access to finance mediates the relationship between financial literacy and firm performance

### Financial literacy, FinTech adoption, and firm performance

As previously noted, SMEs face significant financial constraints, which adversely impact firm performance and growth. FinTech solutions are one of the most basic forms of technology that replace traditional financial services, crucial particularly for SMEs who often struggle with financial limitations. Innovative FinTech solutions, beyond rudimentary advancements, such as peer-to-peer, crowdfunding, and digital lending platforms, provide viable alternatives to address these financing challenges faced by SMES. The adoption of such technologies enables SMEs to access a consistent line of credit more readily and at a lower cost compared to traditional financing methods, reducing financing gaps for firms [4,92]. Integration of these tools in digital finance enables SMEs to optimize their operational processes and cash flow management, improving the general level of performance metrics; hence, it makes FinTech one of the key drivers for SME success [93]. FinTech has been linked with improved performance and firm sales [94–96]. Dhiaf, Khakan [97] found that firms, compared to non-FinTech firms, exhibit superior manufacturing efficiency practices, which positively impact their market performance. FinTech has also been linked to reduced credit constraints, consequently leading to firms’ innovations [98].

It is plausible that advanced financial literacy could mitigate credit and financing constraints for SMEs by facilitating the adoption of FinTech solutions to support firm growth. Financially literate owner-managers, equipped with superior knowledge and awareness of the financial landscape, macroeconomic conditions, and financial markets, are better positioned to leverage sophisticated FinTech innovations. Unlike traditional financial literacy, this advanced form of financial literacy encompasses a deeper understanding of complex financial mechanisms, potentially driving greater adoption of FinTech solutions to enhance access to financing. Financial literacy has been seen with increased FinTech awareness, which may lead to experimenting with FinTech products and services [41,99]. Basic FinTech technologies such as e-banking and e-payments are more likely to be adopted by the financially literate due to enhanced awareness in the financing landscape [41]. Individuals with a strong foundation in financial concepts are better equipped to understand and adopt complex FinTech tools [100]. Financial literacy enhances FinTech adoption by providing the knowledge and confidence needed to use digital financial tools effectively [43,101]. Kakinuma [102] finds that financial literacy does not directly impact FinTech adoption, but individuals with high leisure tend to adopt FinTech as they have more time to experiment with technology. Business owners and managers often lead busy lives, particularly in larger organizations, which may limit their inclination to engage extensively with new technologies. Jünger and Mietzner [103] revealed that financial literacy is less crucial for P2P lending but plays a pivotal role in the adoption of financial advice services in German households. Authors reveal that greater financial education, which often associated with financial literacy, is more likely to adopt FinTech services because of better comprehension of financial products and improved ability to identify superior offerings of FinTech. Setiawan, Nugraha [43] states that individuals with better financial knowledge are more likely to be open to experimenting with FinTech, as they can better assess its benefits and risks. A recent study suggests that FinTech drives financial literacy, which suggests that the relationship can be bidirectional, implying that perceived benefits and ease of FinTech innovations encourage SMEs to develop financial knowledge and skills as a necessity to utilize FinTech [96]..

A major hurdle in comparing these studies arises from their predominant reliance on measuring basic financial literacy. In a business or firm-specific context, this approach often lacks construct validity. The rationale is that a fundamental comprehension of financial and economic concepts, while seemingly intuitive, does not necessarily correlate with, or translate into, demonstrably useful or materially significant outcomes within complex organizational settings [44]. For instance, work by Nugraha, Setiawan [44] utilizes traditional financial literacy measurements, which are unlikely to be correlated with FinTech adoption. Current literature provides fragmented insights into the relationship between financial literacy and FinTech adoption. Inconsistent findings and weak effects are observed across a subset of these studies. However, the theoretical underpinnings offered to explain these non-significant outcomes often lack empirical support. A possible Furthermore, there is a shortage of replication studies that corroborate these non-significant results. While financial literacy may exert varying influences on FinTech adoption depending on specific contexts, its impact might be subtle due to the presence of other significant drivers of FinTech adoption.

H_5_: Financial literacy has a positive impact on FinTech adoption

H_6_: FinTech adoption positively impacts firm performance

H_7_: FinTech adoption mediates the relationship between financial literacy and firm performance

## Methods

### Sample, data collection, and data analysis technique

A deductive approach is utilized and follows the positivist philosophy to achieve the research objectives. The data were collected primarily from SMEs in the Western Province of Sri Lanka using a structured questionnaire. The study focuses on the Western Province, as the region accounts for the largest concentration of SMEs, representing over 40% of the SMEs in Sri Lanka. Moreover, the selected region covers the country’s commercial capital against other provinces (Central Bank of Sri Lanka, 2024). The questionnaire was distributed in person or through online means to the owner or one of the top-level managers of the respective firm, depending on who implements the financial decisions regarding the firm. In Sri Lanka, small and medium-sized enterprises are those with under 300 employees [104]. Small enterprises range from 11 to 50 employees, and medium-sized enterprises employ 51–300 people. Data from foreign-owned SMEs was not collected, as foreign-owned firms, as seen in the literature it are seen to outperform domestic firms [5]. Eligible respondents were owners of locally owned SMEs operating in the Western Province of Sri Lanka who were directly involved in financial decision-making. The study only included firms with 11–300 employees, in line with the national SME definition, and excluded foreign-owned enterprises due to their systematically different performance characteristics. Participation was voluntary, and only respondents who provided informed consent were included. The questionnaire records consent as the first question; if consent is not given, the questionnaire automatically ends. As part of the ethical research process, the confidentiality of participants is ensured, and the data is anonymized.

The questionnaire is divided into six sections. The first section only has one question that records participant consent. The second section gathers information about the business and the participants’ characteristics. The third section assesses financial literacy. The fourth section measures access to finance. The fifth section measures FinTech adoption. The final section measures firm performance. The questionnaire contained only closed-ended questions with a five-point Likert scale (1 = strongly disagree, 5 = strongly agree) to measure respondents’ levels of agreement. There is still an absence of a comprehensive SME database for deriving a sampling frame for Sri Lankan SMEs. Hence, convenience sampling was utilized to collect the data based on availability and ease of access for the participants.

The collected data was analyzed using the Partial Least Squares Structural Equation Modeling (PLS-SEM) technique to test the hypotheses. This is an effective technique for social science scholarly work as it can analyze complex relationships among latent constructs while accounting for measurement error. Unlike ordinary least squares regression or similar methodologies, PLS-SEM does not necessitate normally distributed data and accounts for measurement errors. This characteristic is particularly relevant in real-world contexts, where data frequently exhibit asymmetry or skewness. Additional consideration was considered, which is assessing mediation and moderation effects (in this case, the mediation effects) between the latent constructs. Thus, Structural Equation Modeling (SEM) was employed to efficiently and simply analyze mediation effects. The application SmartPLS 4.1.0.9 was used as an analysis tool, and IBM SPSS Statistics 27 was used to generate descriptives for the data.

### Ethical consideration

To ensure ethical consent, participants were provided with detailed information about the study’s objectives and the questionnaire. All concerns were clarified prior to obtaining consent for participation. Explicit written consent was obtained from each respondent by ticking the consent statement in questionnaire. Participation was entirely voluntary, and only those who explicitly consented were provided with access to the questionnaire. This con-sent process was witnessed by the authors. The study was conducted with the ethical approval of the Sri Lanka Institute of Information Technology (SLIIT) Business School Ethics Review Committee (SLIIT/ERC/SBS/2024/11).

### Measurement of variables

There is no universally accepted scale to measure financial literacy for owners-managers, most authors adopt financial literacy scales that were used to measure individual-level financial literacy. Individual-level scales for measuring financial literacy are impractical for similar studies and lengthen the questionnaire, which can harm the response rate. This study adopted the scale developed and validated by Diéguez-Soto, Martínez-Romero [61] as it is more practical for the present research and tailored in the context of firm-level study. This scale subjectively measures financial literacy and contains six items. Financial literacy is evaluated based on respondents’ subjective understanding and application of financial concepts, economic evolution, and knowledge of the financial landscape.

Similarly to financial literacy, there is no universally accepted scale to measure access to finance as well. This study utilizes items adapted by Adomako, Danso [105] to measure access to finance, which the author cross-referred from Cooper, Fernando [106] and Wiklund and Shepherd [107]. Access to finance was measured using five items, capturing perceptions of the ease of obtaining funding, confidence in securing additional resources, and challenges encountered in financial access. Many of the studies on FinTech adoption used the behavioral intention dimension to measure the construct [43,44,108]. This study adapted items from Nugraha, Setiawan [44] to measure the behavioral intention dimension of FinTech adoption, but slightly modified them to fit the context of the study. Thus, includes three items that measure behavioral intention to use and adopt FinTech products and services such as digital banking, digital lending, crowdfunding platforms, etc., along with the perceived benefits and willingness to continue using these tools. Firm performance is a multifaceted construct with different definitions among scholarly articles. This study adapts the four items used by Mabula and Ping (29), measuring steady firm growth, steady increase in the number of employees, increase in productivity, and the ability to buy fixed assets. The items originated from a World Bank enterprise survey.

### Respondent’s profile and business characteristics

This study collects limited demographic and business-related data from 264 participants (Table 1). The sample size is justified under the 10 times rule recommended by Hair [109]. The sample predominantly comprised male respondents (91%) aged between 45 and 54 years. The majority of participants possessed an average level of education, encompassing individuals with educational attainment ranging from primary to collegiate levels (grades 12 and 13). High education was defined as the completion of bachelor’s degrees or other professional certifications, while exceptional education was categorized as the attainment of master’s degrees, doctoral degrees (Ph.D.), or the possession of multiple academic qualifications. Education levels were classified based on the number and type of qualifications, with scores assigned accordingly. For instance, individuals with multiple bachelor’s degrees were classified as having exceptional education.

**Table 1:** Demographic characteristics and business characteristics.

Demographic/business characteristic	categorization	Frequency and percentage
Business Size	Medium	71 (27%)
	Small	193 (73%)
Years of Operation	Less than 1 year	3 (1%)
	Between 1 and 3 years	34 (13%)
	Between 3 and 5 years	68 (26%)
	Between 5 and 10 years	30 (11%)
	More than 10 years	129 (49%)
Industry Sector	Agriculture	2 (1%)
	Construction	12 (5%)
	Manufacturing	30 (10%)
	Other	70 (27%)
	Other services (health, education services, law services)	31 (12%)
	Transportation and communication	5 (2%)
	Wholesale and retail	114 (43%)
Age	Under 25	8 (3%)
	25-34	34 (13%)
	35-44	55 (21%)
	45-54	115 (43%)
	55 and above	53 (20%)
Gender	Female	23 (9%)
	Male	241 (91%)
Education Level	No education	2 (1%)
	Low education	61 (23%)
	Average education	159 (60%)
	High education	39 (15%)
	Exceptional education	3 (1%)

Notes:

Regarding business characteristics, many enterprises included in the study were small businesses (73%), and a significant proportion had been in operation for more than 10 years (49%). As anticipated, the wholesale and retail sectors represented the dominant industry among the participating enterprises (43%), likely due to their relatively low barriers to entry and exit.

1. FL stands for financial literacy, FA stands for FinTech adoption, AF stands for access to finance, and FP stands for firm performance
2. Responses were measured on a five-point Likert scale, where 1 represents ‘Strongly Disagree’, 2 represents ‘Disagree’, 3 represents ‘Neutral’, 4 represents ‘Agree’, and 5 represents ^‘^Strongly Agree’

**Fig 1:**
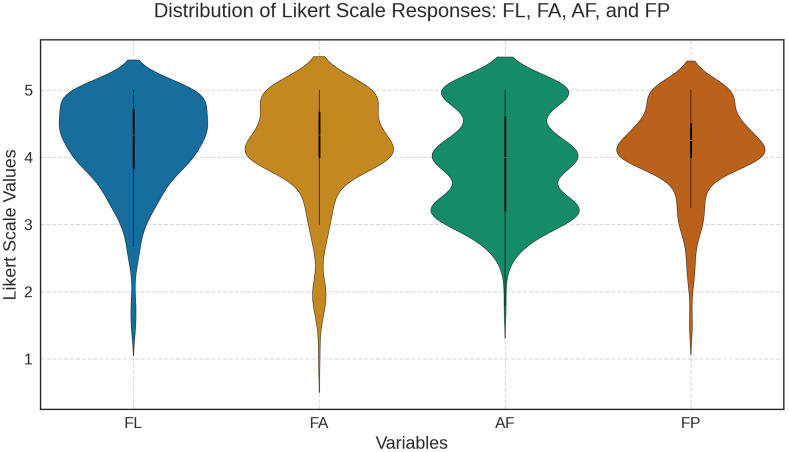
Violin plot of responses.

Fig 1 demonstrates the distribution of the response per construct. Responses for financial literacy appear to be symmetrical with a slight skew toward higher values, suggesting above-average levels of financial literacy among participants. Responses for access to finance items are more spread and clustered around mid-points suggesting some owners and managers struggle with accessing funds while the rest seem to be otherwise. Items of financial literacy and access to finance seem to have a wider range of responses compared to FinTech adoption and firm performance. This suggests that participants have different opinions about those question items, as they are the most complex and subjective items in the survey. Scores for FinTech adoption and firm performance are skewed towards higher values, suggesting participants have positive intentions to adopt FinTech solutions and firms are generally performing well compared to the last year.

## Results

**Fig 2:**
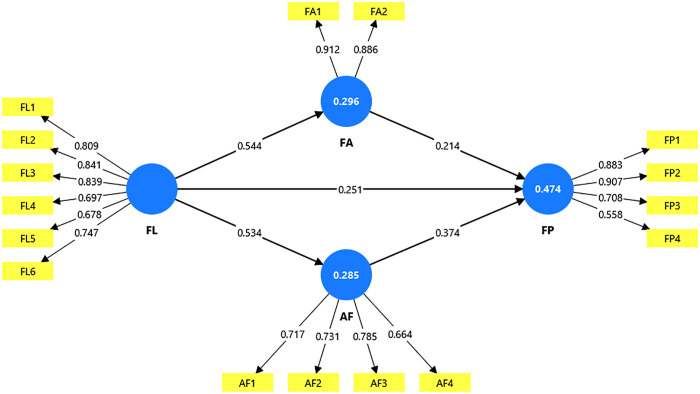
Path diagram of the model.

PLS SEM analysis is a multistep process where the assessment of the measurement model becomes the initial step. The measurement model is illustrated in Fig 2.

### Measurement model

#### Reliability statistics.

Hypothesis tests should follow ensuring adequate construct reliability, discriminant validity, and convergence validity. This study utilizes the Dijkstra–Henseler’s indicator as composite reliability (rho_a), Cronbach alpha, factor loadings, average variance extracted (AVE), and heterotrait-monotrait ratio (HTMT) to assess the reflective construct reliability, convergent validity, and discriminant validity.

Factor loadings demonstrate the variance of an observed variable that can be explained by the factor. An item can be deleted to improve AVE and Composite reliability for the construct [109,110]. All the factor loadings for each reflective construct are satisfactory >0.50 but loadings above 0.70 are desired [109]. One item containing the lowest factor loading was removed from access to the finance construct since the removal of the item improved the AVE of the construct and did not significantly harm the content validity. Hair, Risher [111] state that the threshold for Cronbach alpha and composite reliability (rho_a) should be 0.70 and above. AVE assesses the amount of variance that a construct captures from its indicators relative to the amount of variance due to measurement error. The critical threshold for AVE is above 0.50 [109]. In accordance with the criteria, all the constructs have adequate internal consistency and convergent validity (Table 2).

**Table 2:** Cronbach alpha, CR, and AVE of constructs.

Construct	Item code	Factor loadings	CA	CR	AVE
Financial literacy			0.862	0.868	0.595
	FL1	0.809			
	FL2	0.841			
	FL3	0.839			
	FL4	0.697			
	FL5	0.678			
	FL6	0.747			
Access to finance			0.700	0.711	0.526
	AF1	0.717			
	AF2	0.731			
	AF3	0.785			
	AF4	0.664			
FinTech adoption			0.764	0.771	0.808
	FA1	0.912			
	FA2	0.886			
Firm performance			0.765	0.795	0.603
	FP1	0.883			
	FP2	0.907			
	FP3	0.708			
	FP4	0.558			

Notes:

1. Bold values represent removed items

2. CA stands for Cronbach Alpha, CR is composite reliability and rho_a values were used, and AVE stands for average variance extracted

Discriminant validity refers to the extent to which a construct is truly distinct from other constructs. Typically, the Fornell-Larcker criterion is used to assess discriminant validity. However, HTMT has been empirically validated to be a better marker of discriminant validity than the Fornell-Larcker criterion [112]. Henseler, Ringle [112] state that both 0.90 and 0.85 threshold HTMT levels are appropriate depending on the research context. All the latent constructs meet the HTMT criteria for both thresholds (Table 3).

**Table 3:** Discriminant validity of the constructs (HTMT).

	Access to finance	FinTech adoption	Financial literacy	Firm performance
Access to finance				
FinTech adoption	0.566			
Financial literacy	0.677	0.671		
Firm performance	0.812	0.657	0.697	

### Structural model

The bootstrapping technique was implemented to evaluate hypotheses. Path coefficients denote the level of influence on the variable from another variable. The path coefficients closer to one represent stronger influence and closer to zero represent weaker influence.

The results (Table 4) are assessed for the 95% confidence interval level. The results demonstrate that financial literacy has a positive impact on firm performance (*p* = .001) supporting H_1_, financial literacy has a positive impact on access to finance (*p* = .000) supporting H_2_, financial literacy has a positive impact on FinTech adoption (*p* = .000) supporting H_5_, access to finance has a positive impact on firm performance (*p =* .000) supporting H_3_, and FinTech adoption has a positive impact on firm performance (*p* = .005) supporting H_6_.

**Table 4:** Path coefficients of direct paths.

Relationship	Original sample	Standard deviation	T-statistics	*p-*value	Remarks
FL → FP	0.251	0.084	2.971	.001	Supported
FL → AF	0.534	0.049	10.886	.000	Supported
FL → FA	0.544	0.059	9.225	.000	Supported
AF → FP	0.374	0.055	6.765	.000	Supported
FA → FP	0.214	0.083	2.571	.005	Supported

Notes:

1. Bootstrapping implemented on significance level 0.050, test type is one-tailed at 10000 sub-samples

2. FL: financial literacy, AF: finance, FA: FinTech adoption, and FP: firm performance

### Mediations

To evaluate the hypotheses related to mediations, specific indirect paths were analyzed. According to the results (Table 5) the path from financial literacy to firm performance through access to finance is significant (*p* = .000), and the path from financial literacy to firm performance through FinTech adoption is significant (*p* = .005). If the direct paths are significant and the indirect paths are significant, a partial mediation can be identified [109]. Since the direct path between financial literacy and firm performance is positive and significant, hence access to finance and FinTech adoption partially mediates the relationship between financial literacy and firm performance, supporting H_6_ and H_7_.

**Table 5:** Specific indirect path.

Relationship	Original sample	Standard deviation	T-statistics	*p-*value	95% Bootstrapped CI	Effect Size	Remarks
FL → AF → FP	0.119	0.037	5.339	.000	LCL 0.142, UCL 0.264	0.119	Partial mediation
FL → FA → FP	0.116	0.050	2.346	.010	LCL 0.042, UCL 0.202	0.116	Partial mediation

Notes:

1. FL: financial literacy, AF: finance, FA: FinTech adoption, and FP: firm performance

2. Partial mediation results when the direct relationship is significant with a significant indirect effect

## Discussion

The findings of this study confirm that financial literacy significantly enhances firm performance, supporting the first hypothesis (*β* = 0.251, *p* = .001). Owners and managers with adequate financial literacy exhibit better decision-making, which leads to efficient resource allocation, improved financial management, and reduced risk exposure. This result aligns with Agyapong and Attram [22], Eniola, Entebang [59], who found that financial literacy aids in creating a competitive advantage caused by improved financial decision-making. Thus, financially literate managers and owners are better equipped to make better judgments in financial management and financial risk mitigation of strategic initiatives, as hinted by Ali and Li [113] and simply better at budgeting, investment, and risk management decisions [114]. Furthermore, this finding extends the individual-level observations to the firm context, where financially literate individuals are more proactive and future-oriented in their investments and utilization of resources [17,49,115]. Additionally, it emphasizes the broader organizational impact of financial literacy. In developing contexts like Sri Lanka, where external economic shocks are common, financial literacy enables SMEs to adapt more effectively by planning and mitigating risks. These findings underscore the pivotal role of financial literacy in fostering business success and enabling firms to navigate dynamic market landscapes effectively while mitigating the adverse impacts of economic changes.

Consistent with the trend in literature, financial literacy positively affects access to finance, supporting H_2_ (*β* = 0.534, *p* < .000). SMEs with financially literate owners or managers are more proficient in preparing accurate financial documents, presenting coherent business plans, and engaging effectively with financial institutions, thereby enhancing their creditworthiness [36,116]. Furthermore, this observation reinforces that financial literacy enhances transparency and trust, directly contributing to improved creditworthiness. Our findings align with Adomako, Danso [105], who found that financial literacy helps reduce informational barriers between SMEs and financial institutions. The ability to maintain proper financial records and articulate the viability of business ventures fosters trust and confidence among lenders, ultimately increasing access to credit. This finding corroborates existing literature emphasizing that financial literacy is crucial for bridging gaps in financial inclusion and addressing barriers to credit [2,105,117]. Moreover, this study confirms H_3_ (*β* = 0.544, *p* < .000), showing that access to finance positively impacts firm performance. SMEs with greater access to financial resources can invest in innovative ventures, expand operations, and exploit market opportunities. Beck, Demirgüç-Kunt [118] and Ayyagari, Demirgüç-Kunt [119] similarly highlighted the direct link between access to finance and improved business outcomes, emphasizing that financial constraints remain a critical barrier to SME growth. Additionally, results support H_4_ (indirect effect *β* = 0.374, *p* < .000), which posits that access to finance partially mediates the relationship between financial literacy and firm performance. This mediating effect suggests that financial literacy not only directly influences firm performance but also creates pathways to financial resources. Access to finance has been seen to mediate between financial literacy and the sustainability of firms [1]. As mentioned by Hussain, Salia [36] financial literacy aids in relieving the information asymmetry between borrower and lender. Thus, one of the ways financial literacies improve business outcomes is by enhancing communication between borrowers and lenders. However, the results do not support whether financial literacy has the capacity to influence all types of financing, specifically informal financial access.

The results also validate the fifth hypothesis (*β* = 0.214, *p* < .005), showing that financial literacy significantly influences FinTech adoption. This supports the notion that financially literate SME leaders are more capable of evaluating and adopting modern financial tools, as also observed by Morgan and Trinh [41] and Long, Morgan [42]. Contrary to Setiawan, Phan [120] and Kakinuma [102], who found only minimal effects, our study presents empirical evidence that financial literacy plays a role in the behavioral intention to use FinTech at the firm level. Discrepancy may stem from our use of a business-context-specific financial literacy scale, which better captures the decision-making dynamics of managers. As suggested by Lusardi [46], Lusardi and Mitchell [47], even the simplest wording differences have a significant impact on the content validity when measuring financial literacy, and every financial literacy scale has limitations. However, these results provide evidence that financial literacy improves firms’ ability to integrate suitable technologies, especially in settings with limited infrastructure [121]. Our findings further support H_6_ (*β* = 0.119, *p* < .000), confirming that FinTech adoption significantly boosts firm performance. This is consistent with Lontchi, Yang [96], and Alkhawaldeh, Alhawamdeh [93], who showed that SMEs benefit from digital financial services such as peer-to-peer lending, mobile payments, and invoice financing. These tools reduce SMEs’ reliance on traditional banks and increase agility in responding to market changes. Compared to traditional finance, FinTech services reduce transaction costs and processing delays, which is particularly beneficial for small firms in developing markets. The results also support H_7_ (indirect effect *β* = 0.116, *p* < .010), demonstrating that FinTech adoption partially mediates the relationship between financial literacy and firm performance. This finding expands upon Gomber, Kauffman [122], who stated that digital transformation initiatives yield better outcomes when paired with a strong foundation in financial knowledge. It also resonates with Ozili [123], who linked FinTech adoption to improved efficiency and financial inclusion. In sum, financially literate SME managers are not only more aware of FinTech but are better prepared to evaluate its strategic fit and deploy it effectively.

Overall, this study advances the literature by offering an integrated model that connects financial literacy to firm performance through both access to finance and FinTech adoption. Unlike prior studies that examined these relationships in isolation, our model demonstrates how financial literacy acts as a foundational enabler of both traditional and digital financial access mechanisms. These findings suggest that improving financial literacy among SME owners and managers in developing economies is not merely beneficial. Efforts to promote targeted financial education, especially those that include applied knowledge about digital tools and lending platforms, can help SMEs overcome structural financing barriers and capitalize on new growth opportunities.

Beyond firm-level dynamics, SMEs in developing economies often operate under turbulent conditions of macroeconomic volatility, including inflationary pressures, fluctuating interest rates, credit tightening, and periodic economic shocks, which significantly influence financing and investment decisions [84,124]. In such environments, the strategic value of knowledge, as emphasized by the KBV, becomes highly context-dependent [53,125]. Financial literacy enhances firms’ ability to interpret macroeconomic signals, manage financial uncertainty, and adjust strategic decisions accordingly. Moreover, regulatory frameworks governing banking practices and FinTech adoption shape the extent to which financially literate firms can translate knowledge into performance outcomes. Stringent lending regulations and compliance requirements may restrict access to formal finance, while evolving FinTech regulations influence trust, perceived risk, and adoption decisions [122,123]. Consequently, the positive effects of financial literacy on access to finance, FinTech adoption, and firm performance observed in this study are conditioned by prevailing macroeconomic and regulatory structures.

## Conclusion

This study explores the role of financial literacy in enhancing firm performance, access to finance, and adoption of FinTech in Sri Lankan SMEs. Aligning with KBV theory in literature, this study concludes that financial literacy significantly aids in improving access to finance for firms, boosting firm performance, and financially literate owners and managers are more likely to adopt financial technology. This study also finds partial mediation between financial literacy and firm performance via access to finance and FinTech adoptions. This suggests that the relationship between financial literacy and firm performance could have more possible mediators that should be uncovered in future research. Financial literacy is an overlooked factor within business contexts. The dominant theoretical argument is that financial literacy is attributed to better financial decision-making and affects precursors to successful business outcomes, such as financial access and similar constructs. However, that is a shortsighted perspective as this study provides evidence that financial literacy is related to the adoption of financial technology. There might be many more variables correlated with financial literacy; surprisingly, the literature provides its linkage to strategic initiatives like product development [65]. Since this study finds a significant direct relationship between financial literacy and firm performance, it is plausible that there could be many more uncovered relationships relating to financial literacy. Future research should prioritize this subject area, as addressing low financial literacy among business owners and managers presents a significant opportunity to enhance firm outcomes. While the complexities of this issue may be considerable, the potential benefits for both individual businesses and the broader economy require further investigation.

### Practical implications

This study implies that the composition of adequate owner-manager financial literacy is vital for firm performance, accessing financing, and adopting financial technology. Thus, owner-managers should be self-aware of their competency in financial literacy and take appropriate actions, such as participating in education or training programs, to foster adequate financial knowledge, awareness, and attitude for successful business outcomes. Additionally, providing financial literacy training to staff, particularly the financial management team, is beneficial for fostering informed financial decision-making and enhancing the accuracy of financial reporting. Firms should prioritize financial transparency to secure formal financing, improve creditworthiness, and enhance trust between creditors and stakeholders. This includes consistently creating periodic financial statements following standardized accounting practices. Additionally, business owners and managers should explore FinTech tools that offer alternatives to traditional financing (e.g., crowdfunding, digital lending) to streamline operations, cut costs, and increase efficiency.

Besides, government institutions and educators should prioritize the development of comprehensive and practical financial literacy programs specifically tailored for entrepreneurs and business owners. These programs should extend beyond basic financial management principles and incorporate advanced topics such as navigating financial markets, optimizing investment strategies, and exploring alternative financing options, such as peer-to-peer lending and crowdfunding. Currently, many government-led financial literacy programs, particularly in developing and underdeveloped economies, tend to be rudimentary, primarily focusing on foundational financial concepts. To effectively empower businesses, these programs must be refined to address the specific financial needs and challenges faced by entrepreneurs. Furthermore, these programs should incorporate information on the availability and regulatory landscape of various FinTech products and services within the respective economy, enhancing the awareness of FinTech products and services. In addition, governmental authorities and policymakers should promote FinTech adoption. Governments can offer grants, tax incentives, or technical support to help firms overcome barriers like high costs or a lack of expertise. Moreover, financial institutions should streamline their products and services and market them in a manner that is accessible to individuals with lower financial literacy. This is particularly critical for rural populations, where financial literacy levels may be limited, to enhance understanding and engagement with these offerings.

### Limitations and future research

As noted in the introduction, identifying the factors influencing firm performance is a complex endeavor. Future research should aim to control variables that may confound the observed relationship, such as foreign ownership, industry sector, and owner-manager business experience, as they are likely to distort the findings. Longitudinal studies incorporating control variables or utilizing a research design similar to that employed within Drexler, Fischer [55] with controlled conditions would provide valuable insights into the impact of financial literacy on business outcomes in depth under various conditions. There is a dearth of studies diving deep into the subject; hence, future research should address this issue.

This study did not consider the entrepreneurial orientation of participants, a factor that could substantially influence the observed relationship. Future research should investigate the potential interplay between entrepreneurial orientation and financial literacy. Furthermore, the relationship between financial literacy and FinTech adoption warrants further investigation. Plausibly, this relationship may be bidirectional and mutually influencing. In addition, the judgement sampling technique adopted, was confined to firms located in the Western Province of Sri Lanka. While this region represents a major economic and commercial hub, the use of a non-probability sample and a geographically restricted context may limit the generalizability of the findings to firms operating in other provinces or regions. Future research is therefore encouraged to employ probability sampling methods and a wider geographical coverage through extended data collection, to enhance the external validity of results.

### Statements and declarations

All authors certify that they have no affiliations with or involvement in any organization or entity with any financial interest or non-financial interest in the subject matter or materials discussed in this manuscript.

## Supporting information

S1 File Dataset. (XLSX)
