# Supplementary material for: Does financial literacy matter for firms? An empirical investigation on Sri Lankan SMEs
Source: PLoS One. 2026 Jul 21;21(7):e0354031. doi: 10.1371/journal.pone.0354031 (PMC13387573; doi:10.1371/journal.pone.0354031)

PLOS ONE

Does financial literacy matter for firms? An Empirical Investigation on Sri Lankan SMEs

--Manuscript Draft--

**Manuscript Number:**	PONE-D-25-06685
**Article Type:**	Research Article
**Full Title:**	Does financial literacy matter for firms? An Empirical Investigation on Sri Lankan SMEs
**Short Title:**	Does financial literacy matter for firms? An Empirical Investigation
**Corresponding Author:**	Krishantha Wisenthige, Ph.D<br>Sri Lanka Institute of Information Technology<br>Malabe, Colombo SRI LANKA
**Keywords:**	Financial literacy; Fintech; Access to finance; SME; Firm performance
**Abstract:**	Small and Medium Enterprises (SMEs) are vital to economic growth and job creation, especially in developing countries. However, their potential is not fully utilized due to challenges related to limited financial inclusion, poor resource management, and underdeveloped capital markets. Though financial literacy is well-researched on personal finance, its role as a knowledge resource at the firm level and its impact on firm performance remain underexplored. This study investigates the influence of owner-manager financial literacy on SME performance, focusing on the mediating roles of FinTech adoption and access to finance. By taking 264 SME owner-managers in the Western Province of Sri Lanka, this study found that financial literacy significantly enhances firm performance. As theorized the study supports the argument that financial literacy facilitates sound decision-making regarding financial management, and financial risk mitigation of strategic initiatives, and financial literacy correlates with FinTech adoption. These results underscore the necessity of enhancing financial literacy and promoting FinTech adoption to fully realize the potential of SMEs within emerging economies. The study contributes a concise yet impactful framework for enhancing the performance of SMEs through the strategic application of financial literacy. Based on the study's findings, it is recommended that enterprise owners and managers prioritize achieving an adequate level of financial literacy. Furthermore, governmental institutions should revise their financial literacy programs, reconsidering their design and implementation strategies to effectively engage key personnel within enterprises. Current programs appear underutilized due to deficiencies in depth and practical applicability of knowledge given in them.
**Order of Authors:**	Geesara Kasthuri Arachchi, BBA (Hons) Business Analytics
	Ishan Perera, BBA (Hons) Business Analytics
	Chathurya Gamage, BBA (Hons) Management Information
	Shehara Wijesinghe, BBA (Hons) Management Information
	Krishantha Wisenthige, Ph.D
	Nirmani Dayapathirana, BBA (Hons) Marketing
**Additional Information:**	
**Question**	**Response**
**Financial Disclosure**<br><br>Enter a financial disclosure statement that describes the sources of funding for the work included in this submission. Review the submission guidelines for detailed requirements. View published research articles from *PLOS ONE* for specific examples.	The author(s) received no specific funding for this work.

Powered by Editorial Manager® and ProduXion Manager® from Aries Systems Corporation

This statement is required for submission and **will appear in the published article** if the submission is accepted. Please make sure it is accurate.<br><br>**Funded studies**<br>Enter a statement with the following details:<br>• Initials of the authors who received each award<br>• Grant numbers awarded to each author<br>• The full name of each funder<br>• URL of each funder website<br>• Did the sponsors or funders play any role in the study design, data collection and analysis, decision to publish, or preparation of the manuscript?<br><br>Did you receive funding for this work?	
**Competing Interests**<br><br>Use the instructions below to enter a competing interest statement for this submission. On behalf of all authors, disclose any competing interests that could be perceived to bias this work—acknowledging all financial support and any other relevant financial or non-financial competing interests.<br><br>This statement is required for submission and **will appear in the published article** if the submission is accepted. Please make sure it is accurate and that any funding sources listed in your Funding Information later in the submission form are also declared in your Financial Disclosure statement.<br><br>View published research articles from *PLOS ONE* for specific examples.	The authors have declared that no competing interests exist

**NO authors have competing interests**<br><br>Enter: *The authors have declared that no competing interests exist.*<br><br>**Authors with competing interests**<br><br>Enter competing interest details beginning with this statement:<br><br>*I have read the journal's policy and the authors of this manuscript have the following competing interests: [insert competing interests here]*<br><br>* typeset	
**Ethics Statement**<br><br>Enter an ethics statement for this submission. This statement is required if the study involved:<br><br>• Human participants<br>• Human specimens or tissue<br>• Vertebrate animals or cephalopods<br>• Vertebrate embryos or tissues<br>• Field research<br><br>Write "N/A" if the submission does not require an ethics statement.<br><br>General guidance is provided below. Consult the submission guidelines for detailed instructions. **Make sure that all information entered here is included in the Methods section of the manuscript.**	To ensure ethical consent, participants were provided with detailed information about the study's objectives and the questionnaire. All concerns were clarified prior to obtaining consent for participation. Explicit consent was obtained from each respondent. Participation was entirely voluntary, and only those who explicitly consented were provided with access to the questionnaire. This con-sent process was witnessed by the authors. The study was conducted with the ethical approval of the Sri Lanka Institute of Information Technology (SLIIT) Business School Ethics Review Committee (SLIIT/ERC/SBS/2024/11).

**Format for specific study types**<br><br>**Human Subject Research (involving human participants and/or tissue)**<br>• Give the name of the institutional review board or ethics committee that approved the study<br>• Include the approval number and/or a statement indicating approval of this research<br>• Indicate the form of consent obtained (written/oral) or the reason that consent was not obtained (e.g. the data were analyzed anonymously)<br><br>**Animal Research (involving vertebrate animals, embryos or tissues)**<br>• Provide the name of the Institutional Animal Care and Use Committee (IACUC) or other relevant ethics board that reviewed the study protocol, and indicate whether they approved this research or granted a formal waiver of ethical approval<br>• Include an approval number if one was obtained<br>• If the study involved *non-human primates*, add *additional details* about animal welfare and steps taken to ameliorate suffering<br>• If anesthesia, euthanasia, or any kind of animal sacrifice is part of the study, include briefly which substances and/or methods were applied<br><br>**Field Research**<br><br>Include the following details if this study involves the collection of plant, animal, or other materials from a natural setting:<br>• Field permit number<br>• Name of the institution or relevant body that granted permission	
**Data Availability**<br><br>Authors are required to make all data underlying the findings described fully available, without restriction, and from the time of publication. PLOS allows rare exceptions to address legal and ethical concerns. See the PLOS Data Policy and FAQ for detailed information.	Yes - all data are fully available without restriction

A Data Availability Statement describing where the data can be found is required at submission. Your answers to this question constitute the Data Availability Statement and **will be published in the article**, if accepted.<br><br>**Important:** Stating 'data available on request from the author' is not sufficient. If your data are only available upon request, select 'No' for the first question and explain your exceptional situation in the text box.<br><br>Do the authors confirm that all data underlying the findings described in their manuscript are fully available without restriction?	
**Describe where the data may be found in full sentences. If you are copying our sample text, replace any instances of XXX with the appropriate details.**<br><br>• If the data are **held or will be held in a public repository**, include URLs, accession numbers or DOIs. If this information will only be available after acceptance, indicate this by ticking the box below. For example: *All XXX files are available from the XXX database (accession number(s) XXX, XXX.*).<br>• If the data are all contained **within the manuscript and/or Supporting Information files**, enter the following: *All relevant data are within the manuscript and its Supporting Information files.*<br>• If neither of these applies but you are able to provide **details of access elsewhere**, with or without limitations, please do so. For example:<br><br>*Data cannot be shared publicly because of [XXX]. Data are available from the XXX Institutional Data Access / Ethics Committee (contact via XXX) for researchers who meet the criteria for access to confidential data.*<br><br>*The data underlying the results presented in the study are available from (include the name of the third party*	All relevant data are within the manuscript and its Supporting Information files.

and contact information or URL).<br>• This text is appropriate if the data are owned by a third party and authors do not have permission to share the data.<br><br>* typeset	
Additional data availability information:	

Manuscript
Click here to access/download;Manuscript;Manuscript.docx

Title Page

The Complete Manuscript Title

Does financial literacy matter for firms? An Empirical Investigation on Sri Lankan SMEs

Name and Address of Authors

Mr. Geesara Kasthuri Arachchi
BBA (Hons) Specializing in Business Analytics,
Sri Lanka Institute of Information Technology,
SLIIT Business School,
New Kandy Road, Malabe, Sri Lanka.
Email – bm21111310@my.sliit.lk
ORCID – 0009-0003-6183-0833

Mr. Ishan Perera
BBA (Hons) Specializing in Business Analytics,
Sri Lanka Institute of Information Technology,
SLIIT Business School,
New Kandy Road, Malabe, Sri Lanka.
Email – bm21583346@my.sliit.lk
ORCID – 0009-0004-5248-7743

Ms. Chathurya Gamage
BBA (Hons) Specializing in Management Information Systems,
Sri Lanka Institute of Information Technology,
SLIIT Business School,
New Kandy Road, Malabe, Sri Lanka.
Email – bm21548482@my.sliit.lk
ORCID – 0009-0001-2081-930

Ms. Shehara Wijesinghe
BBA (Hons) Specializing in Management Information Systems,
Sri Lanka Institute of Information Technology,
SLIIT Business School,
New Kandy Road, Malabe, Sri Lanka.
Email – bm21548314@my.sliit.lk
ORCID – 0009-0004-9996-5917

Name and address for correspondence

Krishantha Wisenthige, PhD
Assistant Professor
SLIIT Business School,
Sri Lanka Institute of Information Technology,
New Kandy Road, Malabe, Sri Lanka.

E-mail: krishantha.w@sliit.lk
Tel.: +94(0)11 754 4801 - Ext 4614
Web: https://www.sliit.lk/faculty-of-business/staff/krishantha.w/
ORCID: 0000-0001-8334-6434

Ms. Nirmani Dayapathirana
Instructor,
SLIIT Business School,
Sri Lanka Institute of Information Technology,
New Kandy Road, Malabe, Sri Lanka.
E-mail: nirmani.d@sliit.lk
Tel.: +94(0)11 754 4801 - Ext 4608
Web: https://www.sliit.lk/faculty-of-business/staff/nirmani.d/
ORCID: 0009-0001-2906-7677

Does financial literacy matter for firms? An Empirical Investigation on Sri Lankan SMEs

Abstract

Small and Medium Enterprises (SMEs) are vital to economic growth and job creation, especially in developing countries. However, their potential is not fully utilized due to challenges related to limited financial inclusion, poor resource management, and underdeveloped capital markets. Though financial literacy is a well-researched area at the individual-level, its role as a knowledge resource at the firm level and its impact on firm performance remain underexplored. This study investigates the influence of owner-manager financial literacy on SME performance, focusing on the mediating roles of FinTech adoption and access to finance. By taking 264 SME owner-managers in the Western Province of Sri Lanka, this study found that financial literacy significantly enhances firm performance. As theorized the study finds that financial literacy facilitates sound decision-making, financial management, risk mitigation, and financial literacy drives FinTech adoption. These results highlight the need to enhance financial literacy and promote FinTech adoption to unlock the potential of SMEs in emerging economies. Policymakers and stakeholders should focus on addressing financial infrastructure gaps and regulatory barriers to support growth in the SME sector and the promotion of education programs for entrepreneurs. The findings provide actionable insights for practitioners and policymakers, illustrating how targeted financial literacy initiatives can empower SME owners to leverage FinTech and access financing to drive business performance effectively. This study fills gaps in the literature on how owner-manager financial literacy influences the ability to leverage financial technology and financing for better business outcomes.

Keywords: Financial literacy, FinTech, access to finance, SME, firm performance

Statements and Declarations

All authors certify that they have no affiliations with or involvement in any organization or entity with any financial interest or non-financial interest in the subject matter or materials discussed in this manuscript.

Author Contributions

This research study examines the impact of financial literacy on SME performance, with a focus on the mediating roles of FinTech adoption and access to finance in the context of SMEs in Sri Lanka. The study was conceptualized by GK, leading this process, with GK, IP, CG, and SW contributing to design and execution. Data collection and curation were conducted by GK, IP, CG, and SW. GK and IP undertook data analysis and interpretation, with contributions from CG and SW. The initial manuscript draft was prepared by GK, IP, CG, SW, KW, and ND, with all authors providing critical review, editing, and approval of the final manuscript.

Acknowledgments

The authors affirm that all original ideas, research methodologies, and interpretations presented in this paper are the sole product of human intellectual effort. AI language tools were employed as a stylistic editing tool to improve the readability and professional presentation of the final manuscript.

Conflict of Interest

The authors declare no competing interests.

Ethical consideration

To ensure ethical consent, participants were provided with detailed information about the study’s objectives and the questionnaire. All concerns were clarified prior to obtaining consent for participation. Explicit written consent was obtained from each respondent by ticking the consent statement in questionnaire. Participation was entirely voluntary, and only those who explicitly consented were provided with access to the questionnaire. This con-sent process was witnessed by the authors. The study was conducted with the ethical approval of the Sri Lanka Institute of Information Technology (SLIIT) Business School Ethics Review Committee (SLIIT/ERC/SBS/2024/11).

Funding

The authors did not receive support from any organization for the submitted work.

Data Availability

The data supporting this study's findings are available from the corresponding author upon reasonable request.

Title: Does financial literacy matter for firms? An Empirical Investigation on Sri Lankan SMEs

Abstract

Small and Medium Enterprises (SMEs) are vital to economic growth and job creation, especially in developing countries. However, their potential is not fully utilized due to challenges related to limited financial inclusion, poor resource management, and underdeveloped capital markets. Though financial literacy is well-researched on personal finance, its role as a knowledge resource at the firm level and its impact on firm performance remain underexplored. This study investigates the influence of owner-manager financial literacy on SME performance, focusing on the mediating roles of FinTech adoption and access to finance. By taking 264 SME owner-managers in the Western Province of Sri Lanka, this study found that financial literacy significantly enhances firm performance. As theorized the study supports the argument that financial literacy facilitates sound decision-making regarding financial management, and financial risk mitigation of strategic initiatives, and financial literacy correlates with FinTech adoption. These results underscore the necessity of enhancing financial literacy and promoting FinTech adoption to fully realize the potential of SMEs within emerging economies. The study contributes a concise yet impactful framework for enhancing the performance of SMEs through the strategic application of financial literacy. Based on the study's findings, it is recommended that enterprise owners and managers prioritize achieving an adequate level of financial literacy. Furthermore, governmental institutions should revise their financial literacy programs, reconsidering their design and implementation strategies

to effectively engage key personnel within enterprises. Current programs appear underutilized due to deficiencies in depth and practical applicability of knowledge given in them.

Keywords: Financial literacy, FinTech, Access to finance, SME, Firm performance

Introduction

Given that SMEs constitute the backbone of most global economies, a robust SME sector is indispensable for sustained and steady economic growth [1-3]. Thus, the determination of factors influencing SME growth and SME performance is imperative to optimize their potential to empower global economies [3]. Firm characteristics, firm strategy, public support, and macroeconomic environmental factors are the most influential factors affecting SMEs and these factors can interplay differently in different contexts [4]. While external factors are beyond a firm's control, internal factors such as managerial capabilities are within its influence.

The capabilities of managers play a crucial role in shaping a firm's direction as they are the ones who employ business practices from top to bottom in the firm [5]. Managerial capabilities of managers play a vital role in shaping a firm's strategy, innovation, and overall performance, firms need to constantly adapt and evolve to survive and thrive [6]. These dynamic capabilities include sensing opportunities, seizing them, and transforming the organization to capitalize on them. In the skillset of a manager, this study suggests that financial literacy should be a foundational skill.

From the global perspective 39% of adults have financial literacy of adults scores minimum financial literacy and overall financial literacy aren’t that great either [7]. Financial literacy has been linked to better personal wealth management and overall improvement in financial decision-making on an individual-level [8-11]. Financial literacy has been linked to superior financial well-

being as well [11]. Despite the dominant literature on financial literacy and personal finance, there is a lack of studies on financial literacy at the firm level and its contribution to the organization as a knowledge resource. However, in the last few years, scholarly attention has surged in the SME research domain. Empirical investigations have suggested financial literacy linkage to technological innovations, growth, performance, and technological adaptations of SMEs [12-15].

Studies suggest that owner-managers financial illiteracy demonstrated a lack of attention to the financing of a firm [16]. Not prioritizing optimal financing for a firm is a grave error that the management of a firm should strive to avoid as financing has a lasting impact on the performance of a firm [17]. Financial constraints poses a particularly formidable barrier to SME growth within developing countries that limits the growth and performance of SMEs [18-20]. Lack of, or if not the inability to meet short-term and long-term debt is one of the key drivers of SME failure [21]. Adequate financial resources empower SMEs to take strategic initiatives such as business expansion, technology adoption, innovations, and R&D [22, 23]. Due to undeveloped capital markets in developing countries, SMEs find difficulties financing their business at reasonable prices [24]. Following the COVID-19 pandemic, central banks have implemented interest-rate hikes to combat inflationary pressures [25]. This has resulted in increased borrowing costs for businesses due to higher interest rates on loans and lenders have become more risk-averse, leading to tighter credit conditions and further escalating borrowing costs, particularly for small and medium-sized enterprises [25]. Therefore, optimal financial decision-making has become more critical than ever, as financial markets are increasingly unforgiving. Most Asian SMEs struggle to participate in capital markets due to information asymmetry [26]. Hussain, Salia (27) argued that financial literacy strongly contributes to resolving the information asymmetry between lenders and borrowers. Financial literacy has been a promising element in elevating financial exclusion to SMEs as it has

been linked to relieving financial constraints for SMEs in both developing and developed economies [1, 27-29].

On the other hand, firms do not necessarily need to rely on traditional banking. Financial technology (FinTech) offers alternative solutions to traditional financial services, such as peer-to-peer lending, mobile money, and peer-to-peer lending, transforming the business landscape rapidly. Traditional banking services fall short due to heavy regulations and long processes when assessing a borrower. FinTech lenders on the other hand can analyze borrowers' information more efficiently and offer loans to people who might not qualify for traditional bank loans [30]. FinTech reduces shortcomings of traditional financial services such as assessing creditworthiness and increases the performance of SMEs [31]. The question arises in this case the composition of financial awareness by the financially literate managers, are they more likely to adopt technologies like FinTech into their firm? Studies suggest that the financial literate embody increased awareness of FinTech products [32-34], while some demonstrate financial literacy does not have a direct impact on FinTech adoptions [35]. Building upon that knowledge this study aims to investigate FinTech adoption and the financial literacy relationship at a firm level. This is another underexplored area that has limited knowledge in the current literature.

The purpose of this study is to assess the impact of owner-manager financial literacy on access to finance, and FinTech adoption in shaping SME performance. This study also complements financial literacy's direct impact on firm performance which has been suggested in the literature [2, 13, 36]. There are a limited number of studies evaluating these factors in combinations in emerging economies. Especially the relationship between financial literacy and FinTech adoption is inconclusive and mixed. This study aims to create a framework for relieving financial constraints through the stimulation of financial literacy for SMEs. This study uses a tailored measurement

instrument to reflect the financial literacy of owner-managers designed by Diéguez-Soto, Martínez-Romero (37) which is more appropriate for measuring in business contexts.

This study makes a significant contribution by providing insights into the role of financial literacy as a knowledge resource at the firm level. Firstly, this study examines the direct relationship between owner-manager financial literacy and FinTech adoption as this relationship is inconclusive in the literature. Secondly, this study investigates the owner-manager financial literacy relationship in accessing funds for the firm complementing the findings of previous studies. Thirdly, this study investigates the direct relationship between financial literacy and firm performance as well as the mediating roles of FinTech adoption and access to finance in the said relationship which has limited knowledge in the literature.

The paper is structured as follows, section 2 provides the current knowledge in the literature and the theoretical grounds for the hypotheses, section 3 contains methodological approaches used in the study, section 4 contains the results, section 5 contains the discussions related to the findings, and lastly section 6 contains the concluding statements, implications, limitations and future research.

Literature review, theory, and research hypotheses

The literature review was conducted through a systematic approach to identify relevant studies and theoretical frameworks that explore the relationship between financial literacy, access to finance, FinTech adoption, and SME performance. Journal articles were carefully analyzed to understand existing research gaps and key findings. This review guided the development of the theoretical model and research hypotheses by integrating insights from prior studies and aligning them with the context of SMEs in emerging economies. The focus was to establish a robust foundation that

links financial literacy to firm performance while considering the mediating roles of access to finance and FinTech adoption.

Financial literacy and firm performance

As per Remund (38), financial literacy encompasses the knowledge, skills, and confidence necessary to manage personal finances effectively, including budgeting, saving, investing, and debt management. Aligning with this idea, financial literacy has been linked to better personal wealth management such as improved stock-market participation, which exerts effects on savings behavior and borrowing behavior practices [8-10, 39, 40]. Low financial literacy has also been linked to suboptimal financial behavior, such as inadequate retirement planning, and carrying debt all the way to retirement [41, 42]. Contributing to that point, the financially illiterate tend to obtain higher interest-rate loans [43]. Therefore, the financial literature is equipped with enhanced risk assessments of financial options when it comes to financial decision-making.

While a substantial body of research exists on the relationship between individual financial literacy and personal finance management [8, 39, 40], significantly less attention has been devoted to understanding the extent to which these competencies translate to effective financial decision-making within the context of businesses' financial management. The prevalent theoretical explanation for the link between financial literacy and firm performance and related constructs centers on a resource-based view [13], but this study utilizes the Knowledge-Based View (KBV) theory. KBV can be identified as the most appropriate theoretical framework for examining the relationship between financial literacy and firm performance or firm growth as it narrows down the focus. According to KBV, the ability to create, integrate, and apply knowledge is critical to gaining and

sustaining a competitive advantage [44, 45] and aligning knowledge resources with strategic requirements is paramount [45].

Financial literacy empowers individuals and organizations by providing the necessary knowledge base for informed financial decision-making, effective resource management, and the alignment of financial strategies with overall business objectives. This synergistic relationship between financial literacy and strategic alignment is crucial for successful business execution. Financial literacy can be identified as a knowledge element that can be utilized to improve financial behavior and financial decision-making. Drexler, Fischer (46) found that micro-entrepreneurs who received simplified, rules of thumb training showed significant improvements in managing their financing. Participants who received the training demonstrated a significant increase in sales during periods of economic downturn for the firm. Furthermore, they exhibited a greater likelihood of maintaining financial records, a crucial aspect for assessing the overall financial health of their businesses. Early work by Eniola, Entebang (40), and Eniola and Entebang (47) posited that financially literate owner-managers contribute significantly to improved firm performance. They argued that this impact stems from the ability of financially literate entrepreneurs to make informed decisions regarding resource procurement, allocation, and utilization to develop and implement effective financial strategies. Diéguez-Soto, Martínez-Romero (37) add to the argument that the presence of financial knowledge enhances financial behavior and therefore leads to an increase in the pursuit of investment opportunities leading to better firm growth. A substantial amount of literature supports that possession of financial literacy is correlated with better firm sustainability, performance, and growth by agreeing that financial literacy leads to superior decision-making [1, 13, 27, 37, 48-50].

Financial knowledge is undeniably crucial but the financial attitude that comes with financial literacy toward financing, risk-taking, and business challenges is important in shaping business outcomes [47]. However, the positive impacts of financial literacy extend beyond merely improving financing outcomes. Financial literacy has been surprisingly linked with better innovation capability for firms [51, 52]. Financial literacy has been linked to successful product development and sustainable competitive performance stating that financially literate managers are better equipped to make sound judgments regarding financial viability when developing new products and resource allocations [53]. Therefore, financially literate managers are better equipped to conduct risk assessments and informed cost assessments regarding firms' strategic decisions.

While financial literacy can be a valuable tool, the presence of financial literacy may not translate into firm growth in all contexts. Its impact can vary across different contexts, and other factors may play a more significant role, especially in developing and underdeveloped economies, as other factors could diminish the importance of financial literacy since macroeconomic factors can drive firm performance [4, 47]. Hence further complementation to the literature on the contribution of financial literacy to business outcomes in various contexts would be valuable. Anticipating the trend in the literature therefore develops the first hypothesis.

H_1: Financial literacy has a positive impact on firm performance

Financial literacy, access to finance, and firm performance

Among numerous hurdles that limit firm growth and performance, financial constraints are a major hurdle [20]. External financing is a catalyst for innovation [54-56], and financial deepening can encourage firm incorporation, unlocking benefits like risk diversification and limited liability [57]. SMEs are more likely to be credit-constrained than larger firms [58]. Financial access is especially

critical for younger SMEs [59]. One key issue for firms when accessing external finance is information asymmetry which is also the foundation of the pecking order theory [60].

Investors and lenders rely heavily on their assessment of a firm's value before committing capital. A fundamental assumption underlying this process is that the firm's management possesses a more comprehensive understanding of the firm's true worth compared to external parties [60]. Information asymmetry could affect the supply-side during financial transactions, where lenders possess less insight into the borrowers' repayment capability and details of their businesses' financial health. This may lead to financial institutions perceiving SMEs as risky choices for lending funds tightening lending terms and elevating interest rates. SMEs could often face supply-side constraints due to high interest rates and collateral requirements [61].

Lack of financial literacy leads to maintaining poor financial transparency posing a challenge for lending institutions in assessing creditworthiness, consequently marginalizing SMEs from formal finance [62-64]. Micro, SMEs are specifically are struggling to maintain financial transparency as they are less likely to keep financial records which contributes to poor financial communication [64-67]. Financial literacy can be attributed as a step forward, especially in reducing the information asymmetry between borrowers and lenders [27]. Financially literate are more likely to understand debt information, debt reporting [68] and more likely to keep financial records [46]. Thus, financial literacy can be considered as one of the factors that could alleviate information asymmetry stimulating better financial access.

The argument is that individuals with higher financial literacy are better equipped to comprehend financial information pertaining to debt products and services. This enhanced understanding is hypothesized to lead to increased utilization of these financial products and services, while simultaneously facilitating more effective communication of the firm's value to external funding

sources. Supporting that argument financial literacy programs have been seen to increase financial inclusion [69]. Individuals who possess financial literacy are seen to be less likely to be excluded financially [70, 71]. Additionally, Singh (72) has found that financial literacy is linked to the usage of finances as well, the author identified that many rural individuals are unaware of how to properly utilize credit services, leading to poor decision-making and reluctance to borrow from formal institutions. Kodongo (73) finds that financial literacy has also been associated with improved access to formal financing for individuals. However, Candiya Bongomin, Munene (74) reveal that not all components of financial literacy correlate with financial inclusion but only the financial attitude. Thus, financial literacy may foster a propensity for individuals to actively seek out financial opportunities, thereby increasing their engagement within financial markets. Furthermore, enhanced financial literacy may facilitate more informed and discerning evaluations of available financial options. There is a dominant body of literature investigating financial literacy's positive effect on accessing finance and broader constructs such as financial inclusion providing evidence to the said argument [1, 12, 29, 50, 75]. Based on this understanding, this study hypothesizes that business owners or managers with higher levels of financial literacy are more likely to successfully secure funding for their ventures.

H_2: Financial literacy has a positive impact on access to finance

H_3: Access to finance positively impact firm performance

H_4: Access to finance mediates the relationship between financial literacy and firm performance

Financial literacy, FinTech adoption, and firm performance

FinTech solutions are one of the most basic forms of technology that replace traditional financial services. FinTech also known as financial technology streamlines traditional financial operations.

FinTech offers alternative means to financing such as peer-to-peer lending, crowdfunding, mobile payments, and digital wallets that are more accessible, efficient, and generally inexpensive. Integration of these tools in digital finance enables SMEs to optimize their operational processes, and cash flow management, improving the general level of performance metrics; hence, it makes FinTech one of the key drivers for SME success [76]. FinTech has been linked with improved performance and firm sales [77-79]. Dhiaf, Khakan (80) found that firms, compared to non-FinTech firms, exhibit superior manufacturing efficiency practices, which positively impacts their market performance. FinTech also has been linked to reduced credit constraints consequently leading to firms' innovations [81].

Currently, the literature presents mixed conclusions regarding the relationship between financial literacy and FinTech. Financial literacy has been seen with increased FinTech awareness which may drive to experimenting with FinTech products and services [32, 82]. Basic FinTech technologies such as e-banking and e-payments are more likely to be adopted by the financially literate [32]. Similarly, financially literate are more used to FinTech high schoolers and college students [83]. Kakinuma (84) finds that financial literacy does not directly impact FinTech adoption but individuals with high leisure tend to adopt FinTech as they have more time to experiment with technology. Business owners and managers often lead busy lives, particularly in larger organizations, which may limit their inclination to engage extensively with new technologies. Jünger and Mietzner (85) suggest that financial literacy is less crucial for P2P lending but plays a role in the adoption of financial advice services in German households. Setiawan, Nugraha (34) argue that financial literacy is minimally influential in FinTech adoption despite being associated with FinTech adoption, whereas user innovativeness is more important. Most studies investigating the relationship between financial literacy and FinTech adoption focus on individual consumers and

are often fragmented, examining only specific subsets of financial technologies. Moreover, there is a lack of research specifically examining FinTech adoption within the context of firms. A recent study hints that FinTech drives financial literacy which suggests that the relationship can be bidirectional [79]. Nugraha, Setiawan (35) reveal that financial literacy does not directly affect FinTech adoption in Indonesian SMEs.

Current literature provides fragmented insights into the relationship between financial literacy and FinTech adoption, lacking a clear theoretical foundation. While financial literacy may exert varying influences on FinTech adoption depending on specific contexts, its impact might be subtle due to the presence of other significant drivers of FinTech adoption. Nonetheless, theorizes that financially literate individuals possess a greater propensity for experimentation and may be more inclined to explore and adopt innovative FinTech solutions. As FinTech adoption in firms is correlated with firm performance as pointed out previously it could be argued that FinTech adoption could mediate between financial literacy and firm performance.

H_5: Financial literacy has a positive impact on FinTech adoption

H_6: FinTech adoption positively impacts firm performance

H_7: FinTech adoption mediates the relationship between financial literacy and firm performance

Methods

Sample, data collection, and data analysis technique

A deductive approach is utilized and follows the positivism philosophy to achieve the research objectives. The data were collected primarily from SMEs in the Western Province of Sri Lanka

using a structured questionnaire from 10/08/2024 to 30/11/2024. The questionnaire was distributed in person or through online means to the owner or one of the top-level managers of the respective firm depending on who implements the financial decisions regarding the firm. The definition of SMEs follows the guidelines of the Ministry of Industries and Commerce of Sri Lanka, which defines SMEs as having less than 300 employees [86]. Data from Foreign-owned SMEs were not collected as foreign-owned firms as in the literature it is seen to outperform domestic firms [4]. The questionnaire records consent as the first question, if the consent is not given the questionnaire automatically skips the sections recording the variables and goes to the submission section. The confidentiality of participants is ensured, and the data is anonymized.

The questionnaire is divided into 6 sections. The first section only has one question that records participant consent. The second section gathers information about the business and the participant's characteristics. The third section assesses financial literacy. The fourth section measures access to finance. The fifth section measures FinTech adoption. The final section measures firm performance. The questionnaire contained only closed-ended questions with a 5-point Likert scale (1 = strongly disagree, 5 = strongly agree) to measure respondents' levels of agreement. There is still an absence of a comprehensive SME database for deriving a sampling frame for Sri Lankan SMEs. Hence, purposive sampling was utilized to collect the data from SMEs and data set is available in S1 appendix.

The collected data was analyzed using the Partial Least Squares Structural Equation Modelling (PLS-SEM) technique to test the hypotheses. This is an effective technique for social science scholarly work as it can analyze mediations and other complex relationships among latent constructs because unlike ordinary least squares regression or similar methodologies, PLS-SEM does not

necessitate normally distributed data. This characteristic is particularly relevant in real-world contexts, where data frequently exhibit asymmetry or skewness. The application SmartPLS 4.1.0.9 was used as an analyzing tool and IBM SPSS Statistics 27 was used to generate descriptives for the data.

Measurement of variables

There is no universally accepted scale to measure financial literacy for owners-managers, most authors adopt financial literacy scales that were used to measure individual-level financial literacy. Individual-level scales for measuring financial literacy are impractical for similar studies and lengthen the questionnaire which can harm the response rate. This study borrows the scale developed and validated by Diéguez-Soto, Martínez-Romero (37) which is more practical for this study and more tailored toward CEOs and managers. This scale subjectively measures financial literacy and contains 6 items.

Similarly to financial literacy, there is no universally accepted scale to measure access to finance as well. This study utilizes items adapted by Adomako, Danso (75) to measure access to finance which the author originally borrowed from Cooper, Gimeno-Gascon (87) and Wiklund and Shepherd (88). Many of the studies on FinTech adoption used the behavioral intention dimension to measure the construct [34, 35, 89]. This study adapts items from Nugraha, Setiawan (35) to measure the behavioral intention dimension of FinTech adoption. Firm performance is a multifaceted construct with different definitions among scholarly articles. This study adapts the items used by Mabula and Ping (29) measuring steady firm growth, steady increase in the number of employees, increase in productivity, and ability to buy fixed assets. The items originated originally from a World Bank enterprise survey.

Ethical consideration

To ensure ethical consent, participants were provided with detailed information about the study's objectives and the questionnaire. All concerns were clarified prior to obtaining consent for participation. Explicit written consent was obtained from each respondent by ticking the consent statement in questionnaire. Participation was entirely voluntary, and only those who explicitly consented were provided with access to the questionnaire. This con-sent process was witnessed by the authors. The study was conducted with the ethical approval of the Sri Lanka Institute of Information Technology (SLIIT) Business School Ethics Review Committee (SLIIT/ERC/SBS/2024/11).

Respondent's profile and business characteristics

This study collects limited demographic and business-related data from 264 participants (Table 1). The sample size is justified under the 10 times rule recommended by Hair (90). The sample predominantly comprised male respondents (91%) aged between 45 and 54 years. The majority of participants possessed an average level of education, encompassing individuals with educational attainment ranging from primary to collegiate levels (grades 12 and 13). High education was defined as the completion of bachelor's degrees or other professional certifications, while exceptional education was categorized as the attainment of master's degrees, doctoral degrees (Ph.D.), or the possession of multiple academic qualifications. Education levels were classified based on the number and type of qualifications, with scores assigned accordingly. For instance, individuals with multiple bachelor's degrees were classified as having exceptional education.

Regarding business characteristics, many enterprises included in the study were small businesses (73%), and a significant proportion had been in operation for more than 10 years (49%). As anticipated, the wholesale and retail sectors represented the dominant industry among the participating enterprises (43%), likely due to their relatively low barriers to entry and exit. Furthermore, most businesses employed between 1 and 10 employees (49%).

Table 1. Demographic characteristics and business characteristics

Demographic/business characteristic	categorization	Frequency and percentage
**Business Size**	Medium	71 (27%)
	Small	193 (73%)
**Years of Operation**	Less than 1 year	3 (1%)
	Between 1 and 3 years	34 (13%)
	Between 3 and 5 years	68 (26%)
	Between 5 and 10 years	30 (11%)
	More than 10 years	129 (49%)
**Industry Sector**	Agriculture	2 (1%)
	Construction	12 (5%)
	Manufacturing	30 (10%)
	Other	70 (27%)
	Other services (health, education services, law services)	31 (12%)
	Transportation and communication	5 (2%)
	Wholesale and retail	114 (43%)
**Age**	Under 25	8 (3%)
	25-34	34 (13%)
	35-44	55 (21%)
	45-54	115 (43%)
	55 and above	53 (20%)
**Gender**	Female	23 (9%)
	Male	241 (91%)
**Education Level**	No education	2 (1%)
	Low education	61 (23%)
	Average education	159 (60%)
	High education	39 (15%)
	Exceptional education	3 (1%)

The following Figure 1 demonstrates the distribution of the response per construct. Responses for financial literacy appear to be symmetrical with a slight skew toward higher values, suggesting

above-average levels of financial literacy among participants. Responses for access to finance items are more spread and clustered around mid-points suggesting some owners and managers struggle with accessing funds while the rest seem to be otherwise. Items of financial literacy and access to finance seem to have a wider range of responses compared to FinTech adoption and firm performance. This suggests that participants have different opinions about those question items, as they are the most complex and subjective items in the survey. Scores for FinTech adoption and firm performance are skewed towards higher values, suggesting participants have positive intentions to adopt FinTech solutions and firms are generally performing well compared to the last year.

Results

Figure 1. Violin plot of responses

The measurement model describes the relationship between the latent constructs and their indicators. The measurement model was calculated utilizing the PLS-SEM algorithm offered by SmartPLS 4. One item was removed from the model to enhance it, and below, Figure 2 illustrates the path diagram of the measurement model.

Figure 2. Path diagram of the model

Measurement model

Reliability statistics

Hypothesis tests should follow ensuring adequate construct reliability, discriminant validity, and convergence validity. This study utilizes the Dijkstra–Henseler's indicator as composite reliability

(rho_a), Cronbach alpha, factor loadings, average variance extracted (AVE), and heterotrait-monotrait ratio (HTMT) to assess the reflective construct reliability, convergent validity, and discriminant validity.

Factor loadings demonstrate the variance of an observed variable that can be explained by the factor. An item can be deleted to improve AVE and Composite reliability for the construct [90, 91]. All the factor loadings for each reflective construct are satisfactory >0.50 but loadings above 0.70 are desired [90]. One item containing the lowest factor loading was removed from access to the finance construct since the removal of the item improved the AVE of the construct and did not significantly harm the content validity. Hair, Risher (92) state that the threshold for Cronbach alpha and composite reliability (rho_a) should be 0.70 and above. AVE assesses the amount of variance that a construct captures from its indicators relative to the amount of variance due to measurement error. The critical threshold for AVE is above 0.50 [90]. In accordance with the criteria, all the constructs have adequate internal consistency and convergent validity (Table 2).

Table 2. Cronbach alpha, CR, and AVE of constructs

Construct	Item code	Factor loadings	CA	CR	AVE
**Financial literacy**			0.862	0.868	0.595
	FL1	0.808			
	FL2	0.841			
	FL3	0.840			
	FL4	0.698			
	FL5	0.678			
	FL6	0.747			
**Access to finance**			0.700	0.711	0.526
	AF1	0.717			
	AF2	0.731			
	AF3	0.785			
	AF4	0.664			
	AF5	**0.497**			
**FinTech adoption**			0.805	0.822	0.718
	FA1	0.865			
	FA2	0.874			

	FA3	0.801			
**Firm performance**			0.765	0.797	0.604
	FP1	0.884			
	FP2	0.908			
	FP3	0.707			
	FP4	0.556			

Notes:

1. Bold values represent removed items
2. CA stands for Cronbach Alpha, CR is composite reliability and rho_a values were used, and AVE stands for average variance extracted

Discriminant validity refers to the extent to which a construct is truly distinct from other constructs. Typically, the Fornell-Larcker criterion is used to assess discriminant validity. However, HTMT has been empirically validated to be a better marker of discriminant validity than the Fornell-Larcker criterion [93]. Henseler, Ringle (93) state that both 0.90 and 0.85 threshold HTMT levels are appropriate depending on the research context. All the latent constructs meet the HTMT criteria for both thresholds (Table 3).

Table 3. Discriminant validity of the constructs (HTMT)

	**Access to finance**	**FinTech adoption**	**Financial literacy**	**Firm performance**
**Access to finance**				
**FinTech adoption**	0.490			
**Financial literacy**	0.677	0.646		
**Firm performance**	0.812	0.622	0.697	

Structural model

The bootstrapping technique was implemented to evaluate hypotheses. Path coefficients denote the level of influence on the variable from another variable. The path coefficients closer to one represent stronger influence and closer to zero represent weaker influence.

The results (Table 4) are assessed for the 95% confidence interval level. The results demonstrate that financial literacy has a positive impact on firm performance (*p*-value=0.002) supporting H_1, financial literacy has a positive impact on access to finance (*p*-value=0.000) supporting H_2, financial literacy has a positive impact on FinTech adoption (*p*-value=0.000) supporting H_5, access to finance has a positive impact on firm performance (*p*-value=0.000) supporting H_3, and FinTech adoption has a positive impact on firm performance (*p*-value=0.002) supporting H_6.

Table 4. Path coefficients of direct paths

Relationship	Original sample	Standard deviation	T-statistics	*p*-value	Remarks
**FL→FP**	0.241	0.082	2.927	0.002	Supported
**FL→AF**	0.533	0.049	10.904	0.000	Supported
**FL→FA**	0.542	0.060	9.016	0.000	Supported
**AF→FP**	0.381	0.055	6.928	0.000	Supported
**FA→FP**	0.222	0.077	2.870	0.002	Supported

Notes:

1. Bootstrapping implemented on significance level 0.050, test type is one-tailed at 10000 sub-samples
2. FL: financial literacy, AF: finance, FA: FinTech adoption, and FP: firm performance

Mediations

To evaluate the hypotheses related to mediations, specific indirect paths were analyzed. According to the results (Table 5) the path from financial literacy to firm performance through access to finance is significant (*p*-value=0.000) and the path from financial literacy to firm performance through FinTech adoption is significant (*p*-value=0.005). If the direct paths are significant and the indirect paths are significant, a partial mediation can be identified [90]. Since the direct path between financial literacy and firm performance is positive and significant, hence access to finance and FinTech adoption partially mediates the relationship between financial literacy and firm performance supporting H_6 and H_7.

Table 5. Specific indirect path

Relationship	Original sample	Standard deviation	T-statistics	*p*-value	Remarks
**FL→AF→FP**	0.204	0.037	5.552	0.000	Partial mediation
**FL→FA→FP**	0.120	0.047	2.554	0.005	Partial mediation

Notes:

1. FL: financial literacy, AF: finance, FA: FinTech adoption, and FP: firm performance
2. Partial mediation results when the direct relationship is significant with a significant indirect effect

Discussion

The findings of this study confirm that financial literacy significantly enhances firm performance, supporting H_1. Owners and managers with adequate financial literacy exhibit better decision-making, which leads to efficient resource allocation, improved financial management, and reduced risk exposure. This aligns with previous studies indicating that financial literacy directly improves firm performance [94, 95]. Thus financially literate managers and owners are better equipped to make better judgments in financial management and financial risk mitigation of strategic initiatives as hinted by Ali, Li (53). As observed in individual contexts, financially literate individuals tend to be more proactive and take greater initiative in managing their finances [8, 96], including planning for future financial needs. This phenomenon appears to extend to the management of firm finances as well. These findings underscore the pivotal role of financial literacy in fostering business success and enabling firms to navigate dynamic market landscapes effectively while mitigating the adverse impacts of economic changes.

Consistent with the trend in the literature, financial literacy positively affects access to finance, supporting H_2. SMEs with financially literate owners or managers are more proficient in preparing accurate financial documents, presenting coherent business plans, and engaging effectively with

financial institutions, thereby enhancing their creditworthiness [27, 97]. The ability to maintain proper financial records and articulate the viability of business ventures fosters trust and confidence among lenders, ultimately increasing access to credit. This finding corroborates existing literature emphasizing that financial literacy is crucial for bridging gaps in financial inclusion and addressing barriers to credit [75]. Moreover, this study confirms H3, showing that access to finance positively impacts firm performance. SMEs with greater access to financial resources can invest in innovative ventures, expand operations, and exploit emerging market opportunities.Beck, Demirgüç-Kunt (98) and Ayyagari, Demirgüç-Kunt (99) similarly highlighted the direct link between access to finance and improved business outcomes, emphasizing that financial constraints remain a critical barrier to SME growth. Additionally, results support H_4, which posits that access to finance mediates the relationship between financial literacy and firm performance. This mediating effect suggests that financial literacy not only directly influences firm performance but also creates pathways to financial resources, as supported by [29]. Access to finance has been seen to mediate between financial literacy and the sustainability of firms [1]. As mentioned by Hussain, Salia (27) financial literacy aids in relieving the information asymmetry between borrower and lender. Thus, one of the ways financial literacy improves business outcomes is by enhancing communication between borrowers and lenders.

The results validate H_5, which states that financial literacy significantly influences FinTech adoption. Financial literacy enables SME owners and managers to understand and evaluate the benefits of adopting digital financial tools, which can streamline operations, improve efficiency, and enhance customer experience. FinTech solutions, such as mobile payments, digital lending platforms, and accounting software, offer transformative potential for SMEs by reducing costs and improving access to financial services [100]. Previous studies reported minimal direct relationships between

financial literacy and FinTech adoption [34, 84]. However, this study strengthens the argument that financially literate SME owners are more likely to explore and adopt FinTech innovations, as they are better positioned to assess their utility and manage associated risks. Abubakar (101) noted that financial literacy enhances the ability of firms to adopt and optimize technological solutions that address specific business needs. Moreover, this study supports H_6, which highlights the positive impact of FinTech adoption on firm performance. The adoption of FinTech tools allows SMEs to access alternative financing mechanisms, such as crowdfunding, peer-to-peer lending, and invoice financing, which alleviate financial constraints and enhance liquidity [76, 79]. These digital financial services help SMEs reduce their dependence on traditional banking systems, enabling them to respond more agilely to market demands. The ability to leverage FinTech platforms has been shown to enhance overall firm performance, particularly in terms of profitability, scalability, and customer satisfaction. Finally, the findings provide empirical support for H_7, which posits that FinTech adoption mediates the relationship between financial literacy and firm performance. This suggests that financially literate SME owners and managers leverage their knowledge to integrate financial technologies into their operations effectively, resulting in enhanced performance. This mediating role aligns with the findings of Gomber, Kauffman (102), who emphasized that financial literacy enables businesses to capitalize on digital transformation initiatives, thereby unlocking new growth opportunities. Furthermore, the study's results are consistent with prior research highlighting the role of FinTech adoption in improving firm efficiency, reducing transaction costs, and fostering financial inclusion [103].

Overall, this study advances existing literature by providing a comprehensive understanding of the interplay between financial literacy, access to finance, FinTech adoption, and firm performance. It

highlights that financial literacy serves as the foundational capability that facilitates both traditional financial access and digital financial integration, ultimately driving SME success. These findings reinforce the importance of policies and initiatives aimed at improving financial literacy among SME owners and managers. Efforts to promote financial education, particularly in developing economies, can play a transformative role in empowering SMEs to overcome financial barriers and leverage technological advancements for growth.

Conclusion

This study explores the role of financial literacy in enhancing firm performance, access to finance, and adoption of FinTech in Sri Lankan SMEs. Aligning with KBV theory and self-developed theories in the literature this study concludes that financial literacy significantly aids in improving access to finance for firms boosting firm performance, and financially literate owners and managers are more likely to adopt financial technology. This study also finds partial mediations between financial literacy and firm performance via access to finance and FinTech adoptions. This interprets that the relationship between financial literacy and firm performance could have more possible mediators that should be uncovered in future research. Financial literacy is an overlooked factor within business contexts, the dominant theoretical argument is that financial literacy is attributed to better financial decision-making and affects precursors to successful business outcomes such as financial access and similar constructs. However, that is a shortsighted perspective as this study provides evidence that financial literacy is related with the adoption of financial technology. There might be many more variables correlated with financial literacy, surprisingly the literature provides its linkage to strategic initiatives like product development [53]. Since this study finds a significant direct relationship between financial literacy and firm performance it is plausible that

there could be many more uncovered relationships relating to financial literacy. Future research should prioritize this subject area, as addressing low financial literacy among business owners and managers presents a significant opportunity to enhance firm outcomes. While the complexities of this issue may be considerable, the potential benefits for both individual businesses and the broader economy require further investigation.

Practical implications

This study implies that the composition of adequate owner-manager financial literacy is vital for firm performance, accessing financing, and adopting financial technology. Thus owner-managers should be self-aware of their competency in financial literacy and take appropriate actions such as participation in education programs or training programs to foster adequate financial knowledge, awareness, and attitude for successful business outcomes.

On the other hand, government institutions should prioritize the development of comprehensive and practical financial literacy programs specifically tailored for entrepreneurs and business owners. These programs should extend beyond basic financial management principles and incorporate advanced topics such as navigating financial markets, optimizing investment strategies, and exploring alternative financing options, such as peer-to-peer lending and crowdfunding. Currently, many government-led financial literacy programs, particularly in developing and underdeveloped economies, tend to be rudimentary, primarily focusing on foundational financial concepts. To effectively empower businesses, these programs must be refined to address the specific financial needs and challenges faced by entrepreneurs. Furthermore, these programs should incorporate information on the availability and regulatory landscape of various FinTech products and services within the respective economy enhancing the awareness of FinTech products and services.

Limitations and future research

As noted in the introduction, identifying the factors influencing firm performance is a complex endeavor. Future research should aim to control for variables that may confound the observed relationship, such as foreign ownership, industry sector, and owner-manager business experience as they are likely to distort the findings. Longitudinal studies incorporating control variables or utilizing a research design similar to that employed within Drexler, Fischer (46) with controlled conditions would provide valuable insights into the impact of financial literacy on business outcomes in depth under various conditions. There is a lack of studies diving deep into the subject, hence future research should address this issue.

This study did not consider the entrepreneurial orientation of participants, a factor that could significantly influence the observed relationship. Future research should investigate the potential interplay between entrepreneurial orientation and financial literacy. Furthermore, the relationship between financial literacy and FinTech adoption warrants further investigation. It is plausible that this relationship may be bidirectional, with each influencing the other. The use of the purposive sampling method in this study may harm the generalizability of the findings. Future research should try to employ probability sampling techniques as much as possible unless there are practical limitations in reaching the targeted samples as in this case.

Supporting information

Appendix S1 – Data set

References

1. Ye J, Kulathunga K. How Does Financial Literacy Promote Sustainability in SMEs? A Developing Country Perspective. Sustainability. 2019;11(10): 2990. doi:https://www.doi.org/10.3390/su11102990.

2. Agyei SK. Culture, financial literacy, and SME performance in Ghana. Cogent Economics & Finance. 2018;6(1). doi:https://www.doi.org/10.1080/23322039.2018.1463813.

3. Zulu-Chisanga S, Chabala M, Mandawa-Bray B. The differential effects of government support, inter-firm collaboration and firm resources on SME performance in a developing economy. Journal of Entrepreneurship in Emerging Economies. 2021;13(2): 175-195. doi:https://www.doi.org/10.1108/JEEE-07-2019-0105.

4. Ipinnaiye O, Dineen D, Lenihan H. Drivers of SME performance: a holistic and multivariate approach. Small Business Economics. 2017;48(4): 883-911. doi:https://www.doi.org/10.1007/s11187-016-9819-5.

5. McKenzie D, Woodruff C. Business Practices in Small Firms in Developing Countries. Management Science. 2016;63(9): 2967-2981. doi:https://www.doi.org/10.1287/mnsc.2016.2492.

6. Teece DJ. Towards a capability theory of (innovating) firms: implications for management and policy. Cambridge Journal of Economics. 2017;41(3): 693-720. doi:https://www.doi.org/10.1093/cje/bew063.

7. OECD. OECD/INFE 2023 International Survey of Adult Financial Literacy. Paris: OECD Publishing, 2023 39 Available from: https://doi.org/10.1787/56003a32-en.

8. van Rooij M, Lusardi A, Alessie R. Financial literacy and stock market participation. Journal of Financial Economics. 2011;101(2): 449 - 472. doi:https://www.doi.org/10.1016/j.jfineco.2011.03.006.

9. de Bassa Scheresberg C. Financial literacy and financial behavior among young adults: Evidence and implications. Numeracy. 2013;6(2): 1-21. doi:https://www.doi.org/10.5038/1936-4660.6.2.5.

10. Morgan PJ, Long TQ. Financial literacy, financial inclusion, and savings behavior in Laos. Journal of Asian Economics. 2020;68: 101197. doi:https://www.doi.org/10.1016/j.asieco.2020.101197.

11. Bai R. Impact of financial literacy, mental budgeting and self control on financial wellbeing: Mediating impact of investment decision making. PLOS ONE. 2023;18(11): e0294466. doi:https://www.doi.org/10.1371/journal.pone.0294466.

12. Okello Candiya Bongomin G, Mpeera Ntayi J, Munene JC, Akol Malinga C. The relationship between access to finance and growth of SMEs in developing economies: Financial literacy as a moderator. Review of International Business and Strategy. 2017;27(4): 520-538. doi:https://www.doi.org/10.1108/RIBS-04-2017-0037.

13. Agyapong D, Attram AB. Effect of owner-manager's financial literacy on the performance of SMEs in the Cape Coast Metropolis in Ghana. Journal of Global Entrepreneurship Research. 2019;9(1): 67. doi:https://www.doi.org/10.1186/s40497-019-0191-1.

14. Duréndez A, Dieguez-Soto J, Madrid-Guijarro A. The influence of CEO's financial literacy on SMEs technological innovation: the mediating effects of MCS and risk-taking. Financial Innovation. 2023;9(1): 15. doi:https://www.doi.org/10.1186/s40854-022-00414-w.

15. Winarsih, Noviyanti D, Mutoharoh, editors. The Influence of Financial Literacy and Financial Technology on SMEs' Sustainability2024; Cham: Springer Nature Switzerland.

16. Brown RB, Saunders MNK, Beresford R. You owe it to yourself: The financially literate manager. Accounting Forum. 2006;30(2): 179-191. doi:https://www.doi.org/10.1016/j.accfor.2006.03.001.

17. Beck T, Demirgüç-Kunt A. Access to Finance: An Unfinished Agenda. The World Bank Economic Review. 2008;22(3): 383-396. doi:https://www.doi.org/10.1093/wber/lhn021.

18. Wang Y. What are the biggest obstacles to growth of SMEs in developing countries? – An empirical evidence from an enterprise survey. Borsa Istanbul Review. 2016;16(3): 167-176. doi:https://www.doi.org/10.1016/j.bir.2016.06.001.

19. Khan MA. Barriers constraining the growth of and potential solutions for emerging entrepreneurial SMEs. Asia Pacific Journal of Innovation and Entrepreneurship. 2022;16(1): 38-50. doi:https://www.doi.org/10.1108/APJIE-01-2022-0002.

20. Ardic OP, Mylenko N, Saltane V. Access to Finance by Small and Medium Enterprises: a Cross-Country Analysis with A New Data Set. Pacific Economic Review. 2012;17(7): 491-513. doi:https://www.doi.org/10.1111/j.1468-0106.2012.00596.x.

21. Zizi Y, Oudgou M, El Moudden A. Determinants and Predictors of SMEs' Financial Failure: A Logistic Regression Approach. Risks. 2020;8(4): 107. doi:https://www.doi.org/doi:10.3390/risks8040107.

22. Hall BH. Investment and Research and Development at the Firm Level: Does the Source of Financing Matter? National Burea of Economic Research. 1992. doi:https://www.doi.org/10.3386/w4096.

23. David B. Audretsch ART. What's New about the New Economy? Sources of Growth in the Managed and Entrepreneurial Economies. Industrial and Corporate Change. 2001;10(1): 267–315. doi:https://www.doi.org/10.1093/icc/10.1.267.

24. Giang MH, Trung BH, Yoshida Y, Xuan TD, Que MT. The Causal Effect of Access to Finance on Productivity of Small and Medium Enterprises in Vietnam. Sustainability. 2019;11(19): 5451. doi:https://www.doi.org/10.3390/su11195451.

25. OECD. OECD SME and Entrepreneurship Outlook 2023. Paris: OECD Publishing, 2023 Available from: https://doi.org/10.1787/342b8564-en.

26. Yoshino N, Taghizadeh-Hesary F. The Role of SMEs in Asia and Their Difficulties in Accessing Finance. Tokyo: Asian Development Bank Institute, 2018 2018. Report No.: 911 Available from: https://www.adb.org/publications/role-smes-asia-and-theirdifficulties-accessing-finance.

27. Hussain J, Salia S, Karim A. Is knowledge that powerful? Financial literacy and access to finance: An analysis of enterprises in the UK. Journal of Small Business and Enterprise Development. 2018;25(6): 985-1003. doi:https://www.doi.org/10.1108/JSBED-01-2018-0021.

28. García-Pérez-de-Lema D, Ruiz-Palomo D, Diéguez-Soto J. Analysing the roles of CEO's financial literacy and financial constraints on Spanish SMEs technological innovation. Technology in Society. 2021;64: 101519. doi:https://www.doi.org/10.1016/j.techsoc.2020.101519.

29. Mabula JB, Ping HD. Financial Literacy of SME Managers' on Access to Finance and Performance: The Mediating Role of Financial Service Utilization. International Journal of Advanced Computer Science and Applications. 2018;9(9). doi:https://www.doi.org/10.14569/IJACSA.2018.090905.

30. Buchak G, Matvos G, Piskorski T, Seru A. Fintech, regulatory arbitrage, and the rise of shadow banks. Journal of Financial Economics. 2018;130(3): 453-483. doi:https://www.doi.org/10.1016/j.jfineco.2018.03.011.

31. Li X, Ye Y, Liu Z, Tao Y, Jiang J. FinTech and SME' performance: Evidence from China. Economic Analysis and Policy. 2024;81: 670-682. doi:https://www.doi.org/10.1016/j.eap.2023.12.026.

32. Morgan PJ, Trinh LQ. Fintech and financial literacy in Viet Nam. ADBI Working Paper Series. Tokyo: Asian Development Bank Institute (ADBI), 2020 2020. Report No.: 1154 Available from: https://hdl.handle.net/10419/238511.

33. Long TQ, Morgan PJ, Yoshino N. Financial literacy, behavioral traits, and ePayment adoption and usage in Japan. Financial Innovation. 2023;9(1): 101. doi:https://www.doi.org/10.1186/s40854-023-00504-3.

34. Setiawan B, Nugraha D, Irawan A, Nathan R, Zoltan Z. User Innovativeness and Fintech Adoption in Indonesia. Journal of Open Innovation: Technology, Market, and Complexity. 2021;7(3): 1-18. doi:https://www.doi.org/10.3390/joitmc7030188.

35. Nugraha DP, Setiawan B, Nathan RJ, Fekete-Farkas M. Fintech Adoption Drivers for Innovation for SMEs in Indonesia. Journal of Open Innovation: Technology, Market, and Complexity. 2022;8(4): 208. doi:https://www.doi.org/10.3390/joitmc8040208.

36. Hidayat-ur-Rehman I. The role of financial literacy in enhancing firm's sustainable performance through Fintech adoption: a moderated mediation analysis. International Journal of Innovation Science. 2024;ahead-of-print(ahead-of-print). doi:https://www.doi.org/10.1108/IJIS-03-2024-0056.

37. Diéguez-Soto J, Martínez-Romero MJ, Corten M, Michiels A. The impact of the CEO's financial literacy on family SMEs' growth: the moderating role of generational stage. Baltic Journal of Management. 2021;17(1): 89-106. doi:https://www.doi.org/10.1108/BJM-01-2021-0003.

38. Remund DL. Financial Literacy Explicated: The Case for a Clearer Definition in an Increasingly Complex Economy. Journal of Consumer Affairs. 2010;44(2): 276-295. doi:https://www.doi.org/10.1111/j.1745-6606.2010.01169.x.

39. Sayinzoga A, Bulte EH, Lensink R. Financial Literacy and Financial Behaviour: Experimental Evidence from Rural Rwanda. The Economic Journal. 2016;126(594): 1571-1599. doi:https://www.doi.org/10.1111/ecoj.12217.

40. Clark R, Lusardi A, Mitchell OS. Financial knowledge and 401(k) investment performance: a case study. Journal of Pension Economics and Finance. 2017;16(3): 324-347. Epub 2015/11/25. doi:https://www.doi.org/10.1017/S1474747215000384.

41. Lusardi A. Financial literacy: Do people know the ABCs of finance? Public Understanding of Science. 2015;24(3): 260-271. doi:https://www.doi.org/10.1177/0963662514564516.

42. Lusardi A, Mitchell OS, Oggero N. Debt and Financial Vulnerability on the Verge of Retirement. Journal of Money, Credit and Banking. 2020;52(5): 1005-1034. doi:https://www.doi.org/10.1111/jmcb.12671.

43. Lusardi A, Tufano P. Debt literacy, financial experiences, and overindebtedness. Journal of Pension Economics and Finance. 2015;14(4): 332-368. Epub 2015/10/01. doi:https://www.doi.org/10.1017/S1474747215000232.

44. Grant RM. Toward a knowledge-based theory of the firm. Strategic Management Journal. 1996;17(S2): 109-122. doi:https://www.doi.org/10.1002/smj.4250171110.

45. Zack MH. Developing a Knowledge Strategy. California Management Review. 1999;41(3): 125-145. doi:https://www.doi.org/10.2307/41166000.

46. Drexler A, Fischer G, Schoar A. Keeping It Simple: Financial Literacy and Rules of Thumb. American Economic Journal: Applied Economics. 2014;6(2): 1-31. doi:https://www.doi.org/10.1257/app.6.2.1.

47. Eniola AA, Entebang H. SME Managers and Financial Literacy. Global Business Review. 2017;18(3): 559-576. doi:https://www.doi.org/10.1177/0972150917692063.

48. Kulathunga KMMCB, Ye J, Sharma S, Weerathunga PR. How Does Technological and Financial Literacy Influence SME Performance: Mediating Role of ERM Practices. Information. 2020;11(6): 297. doi:https://www.doi.org/10.3390/info11060297.

49. Wahyono, Hutahayan B. The relationships between market orientation, learning orientation, financial literacy, on the knowledge competence, innovation, and performance of small and medium textile industries in Java and Bali. Asia Pacific Management Review. 2021;26(1): 39-46. doi:https://www.doi.org/10.1016/j.apmrv.2020.07.001.

50. Susan M. Financial Literacy and Growth of Micro, Small, and Medium Enterprises in West Java, Indonesia. In: Barnett WA, Sergi BS, editors. Advanced Issues in the Economics of Emerging Markets. International Symposia in Economic Theory and Econometrics. 27: Emerald Publishing Limited; 2020. p. 39-48.

51. Tian G, Zhou S, Hsu S. Executive financial literacy and firm innovation in China. Pacific-Basin Finance Journal. 2020;62: 101348. doi:https://www.doi.org/10.1016/j.pacfin.2020.101348.

52. Liu B, Wang J, Chan KC, Fung A. The impact of entrepreneurs's financial literacy on innovation within small and medium-sized enterprises. International Small Business Journal. 2021;39(3): 228-246. doi:https://www.doi.org/10.1177/0266242620959073.

53. Ali S, Li G, Yang P, Hussain K, Latif Y. Unpacking the importance of intangible skills in new product development and sustainable business performance; strategies for marketing managers. PLOS ONE. 2020;15(9): e0238743. doi:https://www.doi.org/10.1371/journal.pone.0238743.

54. Adegboye AC, Iweriebor S. Does Access to Finance Enhance SME Innovation and Productivity in Nigeria? Evidence from the World Bank Enterprise Survey. African Development Review. 2018;30(4): 449-461. doi:https://www.doi.org/10.1111/1467-8268.12351.

55. Turyakira P, Kasimu S, Turyatunga P, Kimuli SN. The joint effect of firm capability and access to finance on firm performance among small businesses: A developing country perspective. African Journal of Business Management. 2019;13(6): 198-206. doi:https://www.doi.org/10.5897/AJBM2019.8758.

56. Mare DS, de Nicola F, Miguel F. Financial Structure and Firm Innovation: Evidence from around the World. Policy Research Working Paper. Washington, DC: World Bank, 2021 2021. Report No.: 9670 Available from: http://hdl.handle.net/10986/35631.

57. Demirguc-Kunt A, Love I, Maksimovic V. Business environment and the incorporation decision. Journal of Banking & Finance. 2006;30(11): 2967-2993. doi:https://www.doi.org/10.1016/j.jbankfin.2006.05.007.

58. Beck T, DemirgÜÇ-Kunt A, Maksimovic V. Financial and Legal Constraints to Growth: Does Firm Size Matter? The Journal of Finance. 2005;60(1): 137-177. doi:https://www.doi.org/10.1111/j.1540-6261.2005.00727.x.

59. Bönte W, Nielen S. Product Innovation, Credit Constraints, and Trade Credit: Evidence from a Cross-country Study. Managerial and Decision Economics. 2011. doi:https://www.doi.org/10.1002/mde.1546.

60. Myers SC, Majluf NS. Corporate financing and investment decisions when firms have information that investors do not have. Journal of Financial Economics. 1984;13(2): 187-221. doi:https://www.doi.org/10.1016/0304-405X(84)90023-0.

61. Stiglitz JE, Weiss A. Credit Rationing in Markets with Imperfect Information. The American Economic Review. 1981;71(3): 393-410.

62. Amoako GK. Accounting Practices of SMEs: A Case Study of Kumasi Metropolis in Ghana. International Journal of Business and Management. 2013;8(24): p73. doi:https://www.doi.org/10.5539/ijbm.v8n24p73.

63. Zotorvie JST. A study of Financial Accounting Practices of Small and Medium Scale Enterprises (SMEs) in Ho Municipality, Ghana. International Journal of Academic Research in Business and Social Sciences. 2017;7(7): Pages 29-39. doi:https://www.doi.org/10.6007/IJARBSS/v7-i7/3075.

64. Bui NT, Le OTT, Nguyen PTT. Management Accounting Practices Among Vietnamese Small And Medium Enterprise. Asian Economic and Financial Review. 2020. doi:https://www.doi.org/10.18488/journal.aefr.2020.101.94.115.

65. Wadesango OV. The importance of record keeping to the growth of small and medium scale enterprises (SMEs) in Zimbabwe. Corporate Ownership and Control. 2015;12(4): 744-755. doi:https://www.doi.org/10.22495/cocv12i4c7p5.

66. Madurapperuma MW, Thilakerathne PMC, Manawadu IN. Accounting Record Keeping Practices in Small and Medium Sized Enterprise's (SME's) in Sri Lanka. Journal of Finance and Accounting. 2016;4(4): 188-193. doi:https://www.doi.org/10.11648/j.jfa.20160404.14.

67. Mang'ana KM, Hokororo SJ, Ndyetabula DW. An Investigation of the Extent of Implementation of the Financial Management Practices of Agri-SMEs in developing countries: Evidence from Tanzania. Sustainable Technology and Entrepreneurship. 2024;3(1): 100049. doi:https://www.doi.org/10.1016/j.stae.2023.100049.

68. Madeira C, Margaretic P. The impact of financial literacy on the quality of self-reported financial information. Journal of Behavioral and Experimental Finance. 2022;34: 100660. doi:https://www.doi.org/10.1016/j.jbef.2022.100660.

69. Beck T, Demirguc-Kunt A. Small and medium-size enterprises: Access to finance as a growth constraint. Journal of Banking & Finance. 2006;30(11): 2931-2943. doi:https://www.doi.org/10.1016/j.jbankfin.2006.05.009.

70. Akpene Akakpo A, Amidu M, Coffie W, Abor JY. Financial literacy, financial inclusion and participation of individual on the Ghana stock market. Cogent Economics & Finance. 2022;10(1): 2023955. doi:https://www.doi.org/10.1080/23322039.2021.2023955.

71. Okello Candiya Bongomin G, Ntayi JM, Munene JC, Nkote Nabeta I. Social capital: mediator of financial literacy and financial inclusion in rural Uganda. Review of International Business and Strategy. 2016;26(2): 291-312. doi:https://www.doi.org/10.1108/RIBS-06-2014-0072.

72. Singh A. Exploring demand-side barriers to credit uptake and financial inclusion. International Journal of Social Economics. 2021;48(6): 898-913. doi:https://www.doi.org/10.1108/IJSE-04-2020-0234.

73. Kodongo O. Financial Regulations, Financial Literacy, and Financial Inclusion: Insights from Kenya. Emerging Markets Finance and Trade. 2018;54(12): 2851-2873. doi:https://www.doi.org/10.1080/1540496X.2017.1418318.

74. Candiya Bongomin GO, Munene JC, Ntayi JM, Malinga CA. Financial literacy in emerging economies. Managerial Finance. 2017;43(12): 1310-1331. doi:https://www.doi.org/10.1108/MF-04-2017-0117.

75. Adomako S, Danso A, Ofori Damoah J. The moderating influence of financial literacy on the relationship between access to finance and firm growth in Ghana. Venture Capital. 2016;18(1): 43-61. doi:https://www.doi.org/10.1080/13691066.2015.1079952.

76. Alkhawaldeh B, Alhawamdeh H, Al-Afeef M, Al-Smadi A, Almarshad M, Fraihat B, et al. The effect of financial technology on financial performance in Jordanian SMEs: The role of financial satisfaction. Uncertain Supply Chain Management. 2023;11(3): 1019-1030. doi:https://www.doi.org/10.5267/j.uscm.2023.4.020.

77. Hau H, Huang YI, Lin C, Shan H, Sheng Z, Wei LAI. FinTech Credit and Entrepreneurial Growth. The Journal of Finance. 2024;79(5): 3309-3359. doi:https://www.doi.org/10.1111/jofi.13384.

78. Bounie D, Camara Y. Card-sales response to merchant contactless payment acceptance. Journal of Banking & Finance. 2020;119: 105938. doi:https://www.doi.org/10.1016/j.jbankfin.2020.105938.

79. Lontchi CB, Yang B, Shuaib KM. Effect of Financial Technology on SMEs Performance in Cameroon amid COVID-19 Recovery: The Mediating Effect of Financial Literacy. Sustainability. 2023;15(3): 2171. doi:https://www.doi.org/10.3390/su15032171.

80. Dhiaf MM, Khakan N, Atayah OF, Marashdeh H, El Khoury R. The role of FinTech for manufacturing efficiency and financial performance: in the era of industry 4.0. Journal of Decision Systems. 2024;33(2): 220-241. doi:https://www.doi.org/10.1080/12460125.2022.2094527.

81. Lorenz E, Pommet S. Mobile money, inclusive finance and enterprise innovativeness: an analysis of East African nations. Industry and Innovation. 2021;28(2): 136-159. doi:https://www.doi.org/10.1080/13662716.2020.1774867.

82. Morgan PJ, Long TQ. Fintech and financial literacy in the Lao PDR. ADBI Working Paper Series. Tokyo: Asian Development Bank Institute (ADBI), 2019 2019. Report No.: 933 Available from: https://hdl.handle.net/10419/222700.

83. Ha D, Nguyen K. Unlocking financial access in a developing country amidst COVID-19: the impacts of financial literacy and fintech. Journal of the Asia Pacific Economy. 2024: 1-37. doi:https://www.doi.org/10.1080/13547860.2024.2386825.

84. Kakinuma Y. Financial literacy and quality of life: a moderated mediation approach of fintech adoption and leisure. International Journal of Social Economics. 2022;49(12): 1713-1726. doi:https://www.doi.org/10.1108/IJSE-10-2021-0633.

85. Jünger M, Mietzner M. Banking goes digital: The adoption of FinTech services by German households. Finance Research Letters. 2020;34: 101260. doi:https://www.doi.org/10.1016/j.frl.2019.08.008.

86. Lanka MoIS. Annual performance report. Ministry of Industries, 2023 Contract No.: 3 Available from: https://www.industry.gov.lk/web/annual-reports/.

87. Cooper AC, Gimeno-Gascon FJ, Woo CY. Initial human and financial capital as predictors of new venture performance. Journal of Business Venturing. 1994;9(5): 371-395. doi:https://www.doi.org/10.1016/0883-9026(94)90013-2.

88. Wiklund J, Shepherd D. Entrepreneurial orientation and small business performance: a configurational approach. Journal of Business Venturing. 2005;20(1): 71-91. doi:https://www.doi.org/10.1016/j.jbusvent.2004.01.001.

89. Kim C, Mirusmonov M, Lee I. An empirical examination of factors influencing the intention to use mobile payment. Computers in Human Behavior. 2010;26(3): 310-322. doi:https://www.doi.org/10.1016/j.chb.2009.10.013.

90. Hair JF. A Primer on Partial Least Squares Structural Equation Modeling (PLS-SEM): SAGE Publications; 2014.

91. Hair JF, Hult GTM, Ringle CM, Sarstedt M, Thiele KO. Mirror, mirror on the wall: a comparative evaluation of composite-based structural equation modeling methods. Journal of the Academy of Marketing Science. 2017;45(5): 616-632. doi:https://www.doi.org/10.1007/s11747-017-0517-x.

92. Hair JF, Risher JJ, Sarstedt M, Ringle CM. When to use and how to report the results of PLS-SEM. European Business Review. 2019;31(1): 2-24. doi:https://www.doi.org/10.1108/EBR-11-2018-0203.

93. Henseler J, Ringle CM, Sarstedt M. A new criterion for assessing discriminant validity in variance-based structural equation modeling. Journal of the Academy of Marketing Science. 2015;43(1): 115-135. doi:https://www.doi.org/10.1007/s11747-014-0403-8.

94. Eniola A, Entebang H, Law K. Financial literacy and SME firm performance. International Journal of Research Studies in Management. 2015;5(1): 31-43. doi:https://www.doi.org/10.5861/ijrsm.2015.1304.

95. Lusardi A, Mitchell OS. The Economic Importance of Financial Literacy: Theory and Evidence. Journal of Economic Literature. 2014;52(1): 5-44. doi:https://www.doi.org/10.1257/jel.52.1.5.

96. Mitchell OS, Lusardi A. Financial Literacy: Implications for Retirement Security and the Financial Marketplace: Oxford University Press; 2011. Available from: https://doi.org/10.1093/acprof:oso/9780199696819.001.0001.

97. Atkinson A, Messy F-A. Measuring Financial Literacy. Paris: OECD, 2012 Available from: https://doi.org/10.1787/5k9csfs90fr4-en.

98. Beck T, Demirgüç-Kunt A, Martinez Peria MS. Bank Financing for SMEs around the World: Drivers, Obstacles, Business Models, and Lending Practices. Rochester, NY: The World Bank

Development Research Group, Sector FaP; 2008 2008-11-01. Report No.: Contract No.: 4785 Available from: https://ssrn.com/abstract=1312268.

99. Ayyagari M, Demirgüç-Kunt A, Maksimovic V. Formal versus Informal Finance: Evidence from China. The Review of Financial Studies. 2010;23(8): 3048-3097. doi:https://www.doi.org/10.1093/rfs/hhq030.

100. Arner DW, Barberis JN, Buckley RP. The Evolution of Fintech: A New Post-Crisis Paradigm? University of New South Wales Faculty of Law Legal Studies Research Paper Series. 2015;(2016-62): 1-45. doi:https://www.doi.org/10.2139/ssrn.2676553.

101. Abubakar HA. Entrepreneurship development and financial literacy in Africa. World Journal of Entrepreneurship, Management and Sustainable Development. 2015;11(4): 281-294. doi:https://www.doi.org/10.1108/WJEMSD-04-2015-0020.

102. Gomber P, Kauffman RJ, Parker C, Weber BW. On the Fintech Revolution: Interpreting the Forces of Innovation, Disruption, and Transformation in Financial Services. Journal of Management Information Systems. 2018;35(1): 220-265. doi:https://www.doi.org/10.1080/07421222.2018.1440766.

103. Ozili PK. Impact of digital finance on financial inclusion and stability. Borsa Istanbul Review. 2018;18(4): 329-340. doi:https://www.doi.org/10.1016/j.bir.2017.12.003.

Figure 1

Click here to access/download;Figure;Figure 1.png

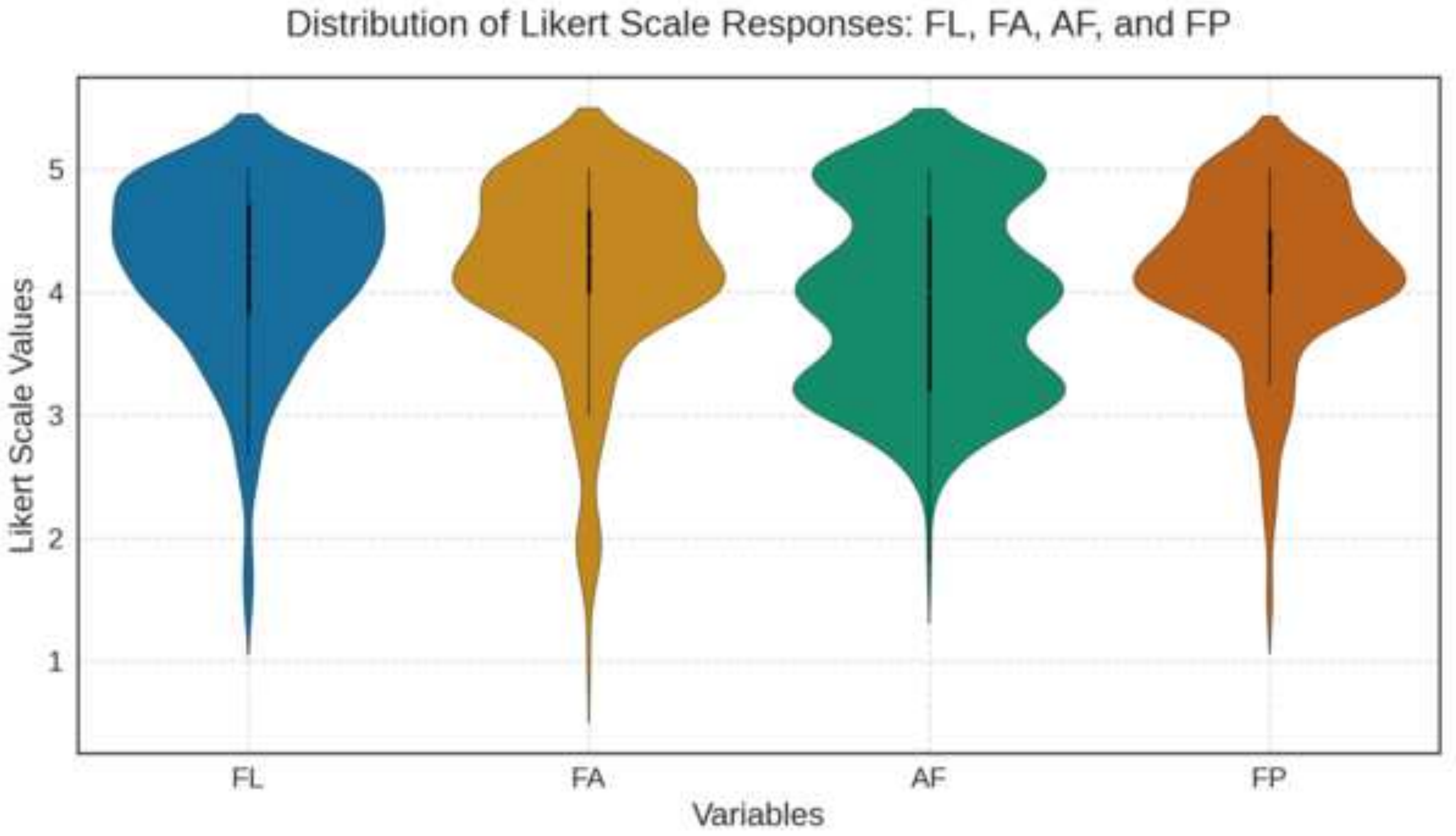

Figure 2

Click here to access/download;Figure;Figure 2.png

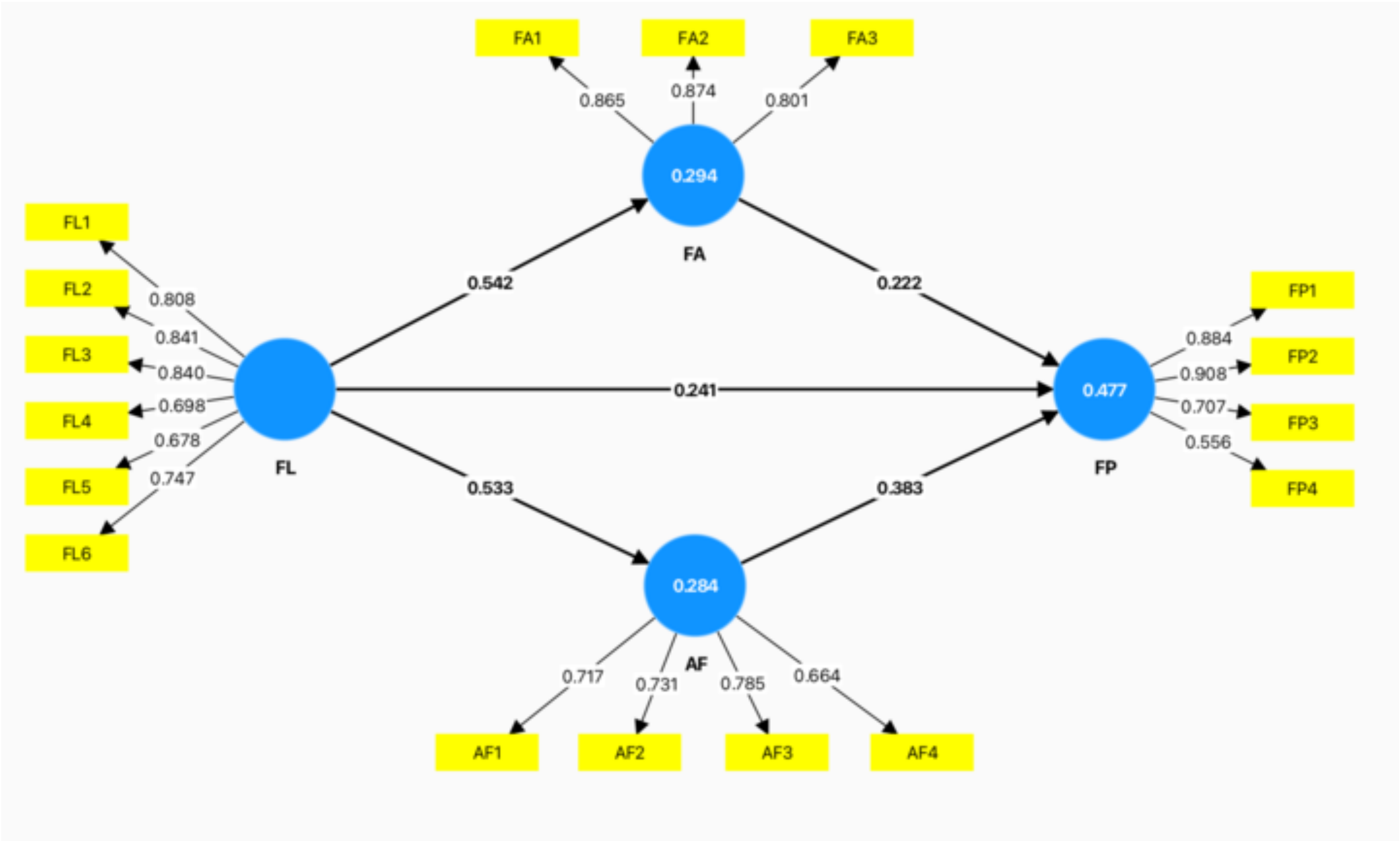

Supplement: Attachment — Submitted filename: PONE-D-25-06685Review.pdf [file pone.0354031.s002.pdf]
