# Supplementary material for: Does financial literacy matter for firms? An empirical investigation on Sri Lankan SMEs
Source: PLoS One. 2026 Jul 21;21(7):e0354031. doi: 10.1371/journal.pone.0354031 (PMC13387573; doi:10.1371/journal.pone.0354031)
Supplement: Attachment — Submitted filename: PONE-D-25-06685 (Review Article).pdf [file pone.0354031.s003.pdf]

Manuscript number : PONE-D-25-06685

Manuscript titled: “Does financial literacy matter for firms? An Empirical Investigation on Sri Lankan SMEs”

Author(s): Krishantha Wisenthige, Ph.D

==

Below is a comprehensive evaluation of the manuscript, spanning from the title to the references, including specific points for improvement that the authors should address, followed by a summary and final decision regarding the article’s acceptance.

1. **Abstract**

- **Issue**:
    - The abstract provides a satisfactory overview but lacks specificity regarding the study’s findings. Statements like "financial literacy significantly enhances firm performance." are not substantiated with quantitative details (e.g., coefficient values or effect sizes).
    - Many confusing statement like “As theorized the study supports the argument that financial literacy facilitates sound decision-making regarding financial management,and financial risk mitigation of strategic initiatives, and financial literacy correlates with FinTech adoption” (e.g, for finding, for suggest statement finding or recommendation)
    - The sample size (254 SMEs) is not explicitly mentioned, despite its importance for contextual understanding.

2. **Introduction**

- **Issue**:
    - The introduction effectively highlights the significance of SMEs and their financing challenges, supported by relevant statistics. However, it fails to explicitly articulate the research gap. What distinguishes this study from prior research?
    - Link this gap to a justification for the study’s importance.

3. **Literature Review**

- **Issue**:
    - Several references are outdated, such as the definition of financing literacy from Remund (2010). In a rapidly evolving field like financial literacy, more recent sources would enhance relevance.

4. **Hypothesis Development**

- **Issue**:
    - This section is brief and lacks in-depth theoretical explanation of independent variable, dependent variable and mediation variable.
    - Bolster the hypothesis foundation with additional literature.

5. **Methodology**

- **Issue**:
  - The methodology is generally clear, but details about the questionnaire are incomplete. Provide specifics on the questionnaire, e.g.: "The questionnaire included 10 items on financial literacy, etc
  - The use of PLS-SEM is noted, but there is no rationale for choosing it over multiple linear regression, which is inconsistently referenced in the abstract.

6. **Results and Discussion**

- **Issue**:
  - Statistical results (path coefficients, T-values, p-values) are well-presented, but the discussion lacks depth. There is insufficient comparison with prior studies to position this research within the literature.
  - Explanations of variable relationships are superficial

7. **Conclusion and Recommendations**

- **Issue**:
  - The conclusion adequately summarizes findings, but the recommendations are overly broad and lack actionable specificity for stakeholders.

8. **References**

- **Issue**:
  - Some references are dated for a dynamic topic like financial literacy

9. **Summary and Decision**

The article demonstrates strengths in its relevant research question, robust methodology, and significant findings for the Indonesian SME context. However, several shortcomings must be addressed to meet international journal standards:

1. **Title**: Simplify for clarity.
2. **Abstract**: Provide specific results and sample size.
3. **Introduction**: Articulate a clear research gap.
4. **Literature Review**: Update references and statistics for timeliness.
5. **Hypothesis Development**: Deepen theoretical explanations.
6. **Methodology**: Include questionnaire details and ensure analytical consistency.
7. **Results and Discussion**: Enhance depth and comparisons with prior studies.
8. **Conclusion and Recommendations**: Offer specific, practical suggestions.
9. **References**: Replace outdated sources with current ones.

Based on this evaluation, I recommend that the article be **accepted with major revisions**. The core research is solid and contributes to the literature, but substantial improvements are necessary to enhance clarity, depth, and relevance. Following major revisions, this manuscript has the potential to be publishable.
