# Supplementary material for: Does financial literacy matter for firms? An empirical investigation on Sri Lankan SMEs
Source: PLoS One. 2026 Jul 21;21(7):e0354031. doi: 10.1371/journal.pone.0354031 (PMC13387573; doi:10.1371/journal.pone.0354031)
Supplement: Attachment — Submitted filename: Second Round PLOS.pdf [file pone.0354031.s005.pdf]

Title/Part	Revision or Feedback	Points for Improvement
Does financial literacy matter for firms? An Empirical Investigation on Sri Lankan SMEs	Abstract	The judgment using a sample in the Western Province of Sri Lanka must be explained as a limitation.
	Background	The background has already presented the research urgency, namely, the lack of literature on financial literacy at the SME level; however, studies on financial literacy in developing economies already exist. What, then, constitutes the distinguishing factor of this study?
	Literature and the theoretical grounds for the hypotheses	The Knowledge-Based View (KBV) is an appropriate framework for explaining financial literacy as a strategic knowledge resource.
	Methodological approaches	Methodology Aligned with the Research Objectives<br>PLS-SEM is suitable for complex models, moderate sample sizes, and the testing of mediating effects.
	Results	
	Discussions	The discussion section consistently and logically links the empirical findings with prior literature and the Knowledge-Based View (KBV) framework. However, the discussion remains relatively descriptive and has not yet fully explored potential macroeconomic factors and regulatory aspects.
	Concluding statements, implications, limitations, and future research	The recommendations for SMEs and the government have clear practical and policy implications, particularly regarding the redesign of financial literacy programs and the promotion of FinTech. The study also acknowledges limitations related to the use of control variables, which are suggested as avenues for future research.
	Overall assessment: Good	This article is ready for publication as it is relevant to the SME, finance, and managerial accounting literature. In addition, the article offers clear empirical contributions, although it still has reasonable methodological limitations typical of survey-based studies.
