# Supplementary material for: Does financial literacy matter for firms? An empirical investigation on Sri Lankan SMEs
Source: PLoS One. 2026 Jul 21;21(7):e0354031. doi: 10.1371/journal.pone.0354031 (PMC13387573; doi:10.1371/journal.pone.0354031)
Supplement: Attachment — Submitted filename: Comments_PONE-D-25-06685R1.pdf [file pone.0354031.s006.pdf]

Comments:

**Section**	**Comments**
**Abstract**	Follow APA format for the latest guideline on reporting p-value
**Introduction**	Line 86 – briefly define FinTech to facilitate better compprehension on the terminology.
	Be consistent – SME Performance? SME Growth? Firm Performance?
**Methods**	Line 358, briefly explain why Western Sri Lanka provides “context” representation for the study
	Line 359 – provide a brief explanation of the respondent’s eligibility criteria
	Line 382 – provide support/citation for this statement - This characteristic is particularly relevant in real-world contexts, where data frequently exhibit asymmetry or skewness.
	Line 363- Data from ~~F~~foreign-owned SMEs
	Line 393 – “and more tailored toward CEOs and managers”, please rephrase this to “..tailored in the context of firm-level study.
	Instead of borrowing the scale, use an academic tone such as adopted/adapted etc
	Line 427 – “most businesses employed between 1 and 10 employees (49%)” – Please check this, as it contradicts the definition of small and medium-sized enterprises in Sri Lanka specified in Lines 362 & 363.
	Line 438 – The construct of Fintech adoption is operationalised as Intention to adopt not existing adopters, and supported by the statement in Line 403. However, the operationalisation of fintech adoption as intention is not aligned with the discussion of the LR, nor the problem statement in the Introduction. My argument is that intention to adopt cannot possibly link to the actual performance, as only usage can imply performance, as supported in the literature section.
**Results**	Line 479 – please use standard p-value reporting in Table format, as the latest APA format has different guidelines for Table and in-text reporting
	Researchers are required to provide results for bootstrapping analysis called Confidence Interval Lower Limit and Upper Limit based on the recommendation by Preacher and Hayes (2008) to add the robustness of the significance t-value result. An additional test is also necessary to specify the effect size of the mediation path.
**Conclusion**	Line 567 – “Aligning with KBV theory and self-developed theories in literature” – This is confusing, as the self-developed theory has not been mentioned at all. In fact, researchers must consistently focus on firm-level theory, not individual-level theory.

**Overall**	Overall, this paper requires serious attention for language and structural editing.
	Check the overall format of citations.
