# Supplementary material for: Does financial literacy matter for firms? An empirical investigation on Sri Lankan SMEs
Source: PLoS One. 2026 Jul 21;21(7):e0354031. doi: 10.1371/journal.pone.0354031 (PMC13387573; doi:10.1371/journal.pone.0354031)
Supplement: Attachment — Submitted filename: Response to Reviewers.docx [file pone.0354031.s007.docx]

**Point-by-Point Response to the Reviewers**

Dear Reviewers,

We sincerely thank you for your thoughtful and constructive comments on our manuscript. We greatly appreciate the time and effort you have invested in reviewing our work. Your insights have been instrumental in helping us improve the quality and clarity of the paper. We have carefully considered each comment and have revised the manuscript accordingly. All responses and revisions are detailed point by point in the following document ***(The mentioned line numbers are in the manuscript without track changes document).***

**Reviewer One**

| **Reviewers’ Comment** | **Response to the Comment** |
| --- | --- |
| 1. The author needs to clarify that the company in question refers to SMEs. The author should mention previous research related to the lack of financial literacy literature in developing countries. It can be related to the situation in Sri Lanka, whether it falls into the category of low literacy or not. | Thank you for your suggestions and feedback. We have clarified that the company in question refers to SMEs. The study focuses on a small or medium-sized enterprise operating in Sri Lanka.  We made changes to provide the details on the financial literacy levels of Sri Lanka and other developing countries The following content was added to the introduction in ***lines 47 to 53*** *“From a global perspective, only 39% of adults are able to score a minimum of financial literacy, indicating that overall financial literacy isn’t that great either [10]. Especially in developing countries, Turkey, Mexico, Colombia, Lebanon, and Uruguay exhibit the lowest financial literacy [11]. In the Sri Lankan context, only 57.9% of the population is financially literate [12]. Regardless of the mediocre financial literacy score, it is reported that Sri Lankan entrepreneurs and investors have poor financial literacy [13, 14]. Hence, the likelihood of negative effects of low financial literacy is higher than ever.”*. |
| 2. You can include previous literature that mentions research has been conducted in developed countries but not in developing countries. | Thank you for your suggestion. We added references in ***lines 103 to 104*** to mention that studies in developed countries outweigh compared in developing countries. The following content is in the said lines *“As demonstrated by Goyal and Kumar (43), there are a limited number of studies evaluating these factors in combination with financial literacy in developing economies compared to studies in developed economies, and even extremely low counts of studies in Sri Lanka.”.* |
| 3. In this study, is firm the other word of SME? (in line 236) | Yes, thank you for highlighting, the “firm” refers to SMEs. “Firm performance” was used instead of SME performance because SME performance is not the standard terminology when referring to the performance of SMEs. It is a broader, more inclusive term that applies to organizations of all sizes, and we do not measure any unique indicators of performance unique to SMEs. |
| 4. I think a clear definition is needed, that SME is a definition of a firm. (Lines 374-376) | Thank you for your suggestion. We further elaborated the classification of small and medium enterprises in the revised manuscript. In ***lines 361 to 363***, we added the following content *“In Sri Lanka, small and medium-sized enterprises are those with under 300 employees [100]. Small enterprises range from 11 to 50 employees, and medium-sized enterprises employ 51 to 300 people”*. |

**Reviewer Two**

| ***Abstract*** | | |
| --- | --- | --- |
| **Reviewers’ Comment** | **Response to the Comment** | |
| 1. The abstract provides a satisfactory overview but lacks specificity regarding the study’s findings. Statements like "financial literacy significantly enhances firm performance." are not substantiated with quantitative details (e.g., coefficient values or effect sizes). | Thank you for the feedback. We included specific findings in the abstract and provided the quantitative measures (coefficients and probability values) of the effects of the findings. The following content in the abstract reflects the requested changes *“By taking a sample of 264 SME owner-managers in the Western Province of Sri Lanka, this study found that financial literacy significantly enhances firm performance (β = 0.241, p < 0.01). The findings indicate that financial literacy supports sound financial decision-making and risk mitigation and is positively associated with both FinTech adoption (β = 0.542, p< 0.001) and access to finance (β = 0.533, p < 0.001).”*. | |
| 2. Many confusing statements like “As theorized the study supports the argument that financial literacy facilitates sound decision-making regarding financial management, and financial risk mitigation of strategic initiatives, and financial literacy correlates with FinTech adoption” (e.g, for finding, for suggest statement finding or recommendation) | Thank you for the constructive feedback. We enhanced the abstract to improve the clarity and removed the confusing statements. The following is the enhanced abstract, *“Small and Medium Enterprises (SMEs) are vital to economic growth and job creation, especially in developing countries. However, their potential is not fully utilized due to challenges related to limited financial inclusion, poor resource management, and underdeveloped capital markets. Though financial literacy is well-researched in personal finance, its role as a knowledge resource at the firm level and its impact on firm performance remain underexplored. This study investigates the influence of owner-managers’ financial literacy on SME performance, focusing on the mediating roles of FinTech adoption and access to finance. By taking a sample of 264 SME owner-managers in the Western Province of Sri Lanka, this study found that financial literacy significantly enhances firm performance (β = 0.241, p < 0.01). The findings indicate that financial literacy supports sound financial decision-making and risk mitigation and is positively associated with both FinTech adoption (β = 0.542, p< 0.001) and access to finance (β = 0.533, p < 0.001). These results underscore the necessity of enhancing financial literacy and promoting FinTech adoption to fully realize the potential of SMEs within developing economies. The study contributes a concise yet impactful framework for enhancing the performance of SMEs through the strategic application of financial literacy. Based on the study's findings, it is recommended that enterprise owners and managers prioritize achieving an adequate level of financial literacy. Furthermore, governmental institutions should revise their financial literacy programs, reconsidering their design and implementation strategies to effectively engage key personnel within enterprises. Current programs appear underutilized due to deficiencies in depth and practical applicability of the knowledge given in them.*  ***Keywords****: Financial literacy, FinTech, Access to finance, SME, Firm performance”*. | |
| 3. The sample size (254 SMEs) is not explicitly mentioned, despite its importance for contextual understanding. | Thank you for the suggestion. We have now included the sample size in the abstract for better contextual understanding. The following lines are added in the abstract of the revised manuscript to mention the sample size. “*By taking a sample of 264 SME owner-managers in the Western Province of Sri Lanka, this study found that financial literacy significantly enhances firm performance (β = 0.241, p < 0.01).”*. | |
| ***Introduction*** | | |
| **Reviewers’ Comment** | **Response to the Comment** | |
| 1. The introduction effectively highlights the significance of SMEs and their financing challenges, supported by relevant statistics. However, it fails to explicitly articulate the research gap. What distinguishes this study from prior research? | Thank you for your constructive feedback. We made changes to the introduction to further emphasize the research gap in the literature, noting limitations (contextual limitations, methodological limitations, and knowledge gaps) in prior studies. ***Lines 102 to 117*** highlight the research gap in the literature. The following content addresses the comment in the said location.  “*The purpose of this study is to assess the impact of owner-manager financial literacy on access to finance and FinTech adoption in shaping SME performance. As demonstrated by Goyal and Kumar (43), there are a limited number of studies evaluating these factors in combination with financial literacy in developing economies compared to studies in developed economies, and even extremely low counts of studies in Sri Lanka. Examining the influence of owner-managers' financial literacy on firm performance within the Sri Lankan context is valuable due to the country's well-documented economic challenges and instability. Understanding how financial literacy enables owner-managers to navigate external economic threats is essential for enhancing firm resilience and sustainability in such an environment. The relationship between financial literacy and FinTech adoption is inconclusive and mixed. Scholars have revealed that this relationship is weak and suggests basic understanding of financial literacy may not translate to meaningful effects on FinTech adoption [41, 42]. This study argues that this is due to financial literacy scale differences; traditional and basic financial literacy scales do not capture the financial literacy required in a business environment. In addition, financial literacy on specific FinTech products and services that offer alternative financing options is scarce. This study aims to create a framework for relieving financial constraints through the stimulation of financial literacy for SMEs”.* | |
| 2. Link this gap to a justification for the study’s importance. |  |  |
| ***Literature review*** | | |
| **Reviewers’ Comment** | **Response to the Comment** | |
| 1. Several references are outdated, such as the definition of financing literacy from Remund (2010). In a rapidly evolving field like financial literacy, more recent sources would enhance relevance. | Thank you for the constructive feedback. We understand your concern about outdated references, especially in a rapidly evolving field such as financial literacy. We initially included Remund (2010)'s definition because it is widely recognized and frequently cited in the existing literature, providing a foundational perspective. To address your comment and enhance the relevance and breadth of our discussion, we have now incorporated the definitions from both the OECD and Lusardi and Mitchell. Lines from 140 to 144 operationalize financial literacy in the firm context. The following content is added to address the said comment.  “*Operationalization of financial literacy is a difficult task; many scholars have different interpretations and definitions. The OECD (13) defines financial literacy as having the awareness, knowledge, skills, attitudes, and behaviors to make smart financial choices. Lusardi and Mitch-ell (42) define financial literacy as “people’s ability to process economic information and make informed decisions about financial planning, wealth accumulation, pensions, and debt”.”.* The following references are used for defining financial literacy,.  10. OECD. OECD/INFE 2023 international survey of adult financial literacy. Paris: OECD Publishing, 2023 39 Contract No.: 39.  44. Lusardi A, Mitchell OS. The economic importance of financial literacy: Theory and evidence. Journal of Economic Literature. 2014;52(1):5-44. doi: 10.1257/jel.52.1.5. | |
| ***Hypothesis development*** | | |
| **Reviewers’ Comment** | **Response to the Comment** | |
| 1. This section is brief and lacks in-depth theoretical explanation of independent variable, dependent variable and mediation variable. Bolster the hypothesis foundation with additional literature. | Thank you for your valuable feedback. We have addressed this comment by providing a more in-depth theoretical explanation of the independent, dependent, and mediation variables in the revised manuscript. Additionally, we incorporated recent studies for hypothesis development and to make theoretical arguments. The whole literature review section has been improved in the revised manuscript. | |
| ***Methodology/methods*** | | |
| **Reviewers’ Comment** | | **Response to the Comment** |
| 1. The methodology is generally clear, but details about the questionnaire are incomplete. Provide specifics on the questionnaire, e.g.: "The questionnaire included 10 items on financial literacy, etc | | Thank you for the suggestion. We have now provided more specific details about the questionnaire in the methods section under the measurement of variables subheading, including the number of items for each construct, and the nature of the items is explained. The following content is in the revised manuscript to reflect the necessary changes: *“This study borrows the scale developed and validated by Diéguez-Soto, Martínez-Romero (51), which is more practical for this study and more tailored toward CEOs and managers. This scale subjectively measures financial literacy and contains 6 items. Financial literacy is evaluated based on respondents' subjective understanding and application of financial concepts, economic evolution, and knowledge of the financial landscape.*  *Similarly to financial literacy, there is no universally accepted scale to measure access to finance as well. This study utilizes items adapted by Adomako, Danso (102) to measure access to finance, which the author originally borrowed from Cooper, Gimeno-Gascon (103) and Wiklund and Shepherd (104). Access to finance was measured using 5 items, capturing perceptions of the ease of obtaining funding, confidence in securing additional resources, and challenges encountered in financial access. Many of the studies on FinTech adoption used the be-havioral intention dimension to measure the construct [41, 42, 105]. This study adapted items from Nugraha, Setiawan (42) to measure the behavioral intention dimension of FinTech adoption, but slightly modified them to fit the context of the study. Thus, includes 3 items that measure behavioral intention to use and adopt FinTech products and services such as digital banking, digital lending, crowdfunding platforms, etc., along with the perceived benefits and willingness to continue using these tools. Firm performance is a multifaceted construct with different definitions among scholarly articles. This study adapts the 4 items used by Mabula and Ping (29), measuring steady firm growth, steady increase in the number of employees, increase in productivity, and ability to buy fixed assets. The items originated originally from a World Bank enterprise survey.”.* |
| 2. The use of PLS-SEM is noted, but there is no rationale for choosing it over multiple linear regression, which is inconsistently referenced in the abstract. | | Thank you for your constructive feedback. We only provided a few considerations for the rationale for choosing PLS-SEM. We did not mention any details about the choice of multiple linear regression in the abstract, nor any details elsewhere in the original manuscript. Regardless, we added an improved rationale in the methods section under the subheading sample, data collection, and data analysis technique. The following content was added to address the comment.  “*The collected data was analysed using the Partial Least Squares Structural Equation Modelling (PLS-SEM) technique to test the hypotheses. This is an effective technique for social science scholarly work as it can analyse complex relationships among latent constructs, accounting for measurement error. Unlike ordinary least squares regression or similar methodologies, PLS-SEM does not necessitate normally distributed data and accounts for measurement errors. This characteristic is particularly relevant in real-world contexts, where data frequently exhibit asymmetry or skewness. Additional consideration was considered, which is assessing mediation and moderation effects (in this case, the mediation effects) between the latent constructs. Thus, Structural equation modelling was employed to efficiently and simply analyse mediation effects.*” |
| **Results and Discussion** | | |
| **Reviewers’ Comment** | | **Response to the Comment** |
| 1. Statistical results (path coefficients, T-values, p-values) are well-presented, but the discussion lacks depth. There is insufficient comparison with prior studies to position this research within the literature. | | Thank you for your constructive feedback. We have significantly enhanced the discussion section by providing a more in-depth interpretation of the statistical results. We have also expanded our comparison with prior studies to effectively position our research within the existing literature and highlight its contributions. |
| 2. Explanations of variable relationships are superficial | | We appreciate the constructive feedback. We have revised the explanations of variable relationships to provide a more comprehensive and nuanced understanding, moving beyond superficial descriptions. |
| **Conclusion and Recommendations** | | |
| **Reviewers’ Comment** | | **Response to the Comment** |
| 1. The conclusion adequately summarizes findings, but the recommendations are overly broad and lack actionable specificity for stakeholders. | | Thank you for your feedback. We improved the practical implications and recommendations with the necessary changes.  The following content was incorporated under the practical implications subheading, in addition to the overall improvements, highlighting practical recommendations for business owners, managers, and financial institutions. “*Additionally, providing financial literacy training to staff, particularly the financial management team, is beneficial for fostering informed financial decision-making and enhancing the accuracy of financial reporting. Firms should prioritize financial transparency to secure formal financing, improve creditworthiness, and enhance trust between creditors and stakeholders. This includes consistently creating periodic financial statements following standardized accounting practices. Additionally, business owners and managers should explore FinTech tools that offer alternatives to traditional financing (e.g., crowdfunding, digital lending) to streamline operations, cut costs, and increase efficiency. In addition, governmental authorities and policymakers should promote FinTech adoption. Governments can offer grants, tax incentives, or technical support to help firms overcome barriers like high costs or a lack of expertise. Moreover, financial institutions should streamline their products and services and market them in a manner that is accessible to individuals with lower financial literacy. This is particularly critical for rural populations, where financial literacy levels may be limited, to enhance understanding and engagement with these offerings.”.* |
| **References** | | |
| **Reviewers’ Comment** | | **Response to the Comment** |
| 1. Some references are dated for a dynamic topic like financial literacy | | Thank you for your valuable comment regarding the currency of our references. We have reviewed our references and updated them where appropriate to ensure that the literature cited is as current and relevant as possible, especially for key concepts and recent developments in the field. The following literature has been added to the revised manuscript.  5. Gankandage A, Jayathilaka R. Unveiling the challenges: exploring start-up hurdles faced by small and medium-sized enterprise entrepreneurs in Sri Lanka. Humanities and Social Sciences Communications. 2025;12(1): 454. doi:https://www.doi.org/10.1057/s41599-025-04775-2.  6. Chowdhury EK. Drivers of entrepreneurship development in South Asia: empirical insights and policy recommendations. Journal of Innovation and Entrepreneurship. 2025;14(1): 46. doi:https://www.doi.org/10.1186/s13731-025-00512-w.  7. Syed AA. Unveiling the nexus of shadow economy and economic growth through the lens of Hofstede’s cultural dimensions: a case of emerging nations. Discover Sustainability. 2025;6(1): 173. doi:https://www.doi.org/10.1007/s43621-025-00948-w.  10. CBSL. Financial literacy survey sri lanka key survey findings 2021. Regional Development Department Central Bank of Sri Lanka, 2021 2021. Report No.: Available from: https://www.cbsl.gov.lk/en/news/release-of-financial-literacy-survey-sri-lanka-2021-publication.  11. Tennekoon S, Liyanage C. Impact of financial literacy levels among Sri Lankan investors on investment choices. South Asian Journal of Finance. 2021;1(2). doi:https://www.doi.org/10.4038/sajf.v1i2.35.  12. Kumari RSPD, Dissanayake DHSW, Deshika NPT. The impact of financial literacy on business performance in small and medium enterprises: evidence from Kegalle District in Sri Lanka. International Journal of Accounting and Business Finance. 2021;7(2). doi:https://www.doi.org/10.4038/ijabf.v7i2.95.  42. Goyal K, Kumar S. Financial literacy: A systematic review and bibliometric analysis. International Journal of Consumer Studies. 2021;45(1): 80-105. doi:https://www.doi.org/https://doi.org/10.1111/ijcs.12605.  47. Huston SJ. Financial literacy and the cost of borrowing. International Journal of Consumer Studies. 2012;36(5): 566-572. doi:https://www.doi.org/https://doi.org/10.1111/j.1470-6431.2012.01122.x.  53. Yakob S, Yakob R, BAM H-S, Rusli RZA. Financial literacy and financial performance of small and medium-sized enterprises. The South East Asian Journal of Management. 2021;15(1): 5. doi:https://www.doi.org/10.21002/seam.v15i1.13117.  54. Widdowson D, Hailwood K. Financial literacy and its role in promoting a sound financial system. Reserve Bank of New Zealand Bulletin. 2007;70(2).  62. Devender K, Kafila, Goli G, Shravan M. Bibliometric trends in SMEs financial literacy research from 2021 to 2024. Future Business Journal. 2025;11(1): 69. doi:https://www.doi.org/10.1186/s43093-025-00495-w.  84. Addo SD, Asantey J, Awadzıe DM. The impact of financial literacy on risk propensity mediated by access to finance. Journal of Business Economics and Finance. 2022;11(4): 195-205.  88. Bani Atta AA. Financial technology platforms and enhancing SME financing in Jordan. Discover Sustainability. 2025;6(1): 568. doi:https://www.doi.org/10.1007/s43621-025-01345-z.  96. Khan MTI, Wei LT, Lee XY. Fintech literacy among millennials: The roles of financial literacy and education. Cogent Social Sciences. 2023;9(2): 2281046. doi:https://www.doi.org/10.1080/23311886.2023.2281046.  100. ADB. Asia Small and Medium-Sized Enterprise Monitor 2024: Designing an MSME Ecosystem for Resilient Growth in Asia and the Pacific: Asian Development Bank; 2024.  110. Padi A, Musah A, Blay MW, Okyere DO. Small and Medium Scale Enterprise (SME) Owner financial literacy, entrepreneurial competencies and financial performance: the role of corporate governance. Future Business Journal. 2025;11(1): 160. doi:https://www.doi.org/10.1186/s43093-025-00585-9.  113. Twumasi MA, Jiang Y, Wang P, Ding Z, Frempong LN, Acheampong MO. Does Financial Literacy Inevitably Lead to Access to Finance Services? Evidence from Rural Ghana. Ciência Rural. 2021;52(3): e20210112. doi:https://www.doi.org/10.1590/0103-8478cr20210112.  78. Charfeddine L, Umlai MI, El-Masri M. Impact of financial literacy, perceived access to finance, ICT use, and digitization on credit constraints: evidence from Qatari MSME importers. Financial Innovation. 2024;10(1): 15. doi:https://www.doi.org/10.1186/s40854-023-00557-4.  79. Lenin M, Sreedevi PM, Ajin S, Ananthi R. Perceptions of Credit Access: MSME Owners Versus Bank Employees on MSME Loans. In: Hamdan RK, editor. Sustainable Data Management: Navigating Big Data, Communication Technology, and Business Digital Leadership Volume 2. Cham: Springer Nature Switzerland; 2025. p. 369-378. |
