# Supplementary material for: Does financial literacy matter for firms? An empirical investigation on Sri Lankan SMEs
Source: PLoS One. 2026 Jul 21;21(7):e0354031. doi: 10.1371/journal.pone.0354031 (PMC13387573; doi:10.1371/journal.pone.0354031)
Supplement: Attachment — Submitted filename: Response to Reviewers PLOS One 2nd Round.docx [file pone.0354031.s008.docx]

**Reviewers’ Comments**

- **Reviewer One**

| **Reviewers’ Comment** | **Response to the Comment** |
| --- | --- |
| Follow APA format for the latest guideline on reporting p-value | Thank you for this valuable suggestion. We have carefully revised the manuscript to ensure that all p-values are reported in strict accordance with the latest APA (7th edition) guidelines. Specifically, p-values are now presented in italicised lowercase (p), exact values are reported where appropriate (e.g., p = .032), and threshold reporting is used only when values are smaller than .001 (e.g., p < .001). These revisions have been applied consistently across the Results section, tables, and figures to improve clarity, accuracy, and compliance with APA reporting standards. |
| Line 86 – briefly define FinTech to facilitate better comprehension on the terminology. | Thank you for your valuable suggestion. In response, lines 87–91 have been revised to provide an explanation of FinTech as per your comment,  “*On top of this, firms do not necessarily need to rely on traditional banking. Financial technology (FinTech), defined as the use of digital technologies to deliver financial services such as payments, lending, and financing in a more efficient and accessible manner, offers alternative solutions to traditional financial services, including peer-to-peer lending and mobile money, thereby transforming the business landscape [37]*”. |
| Be consistent – SME Performance? SME Growth? Firm Performance? | Thank you for highlighting the need for consistency. In accordance with your comment, lines 26–135 in the Introduction have been revised to focus on firm performance. |
| Line 358, briefly explain why Western Sri Lanka provides “context” representation for the study | We appreciate the valuable comment provided to enhance the study. In response, we have included the rationale for specifically selecting the Western Province in lines 392–396, as follows:  *“The study focuses on the Western Province, as it accounts for the largest concentration of small and medium-sized enterprises (SMEs), representing over 40% of the total number of SMEs in Sri Lanka. Moreover, it serves as the country’s commercial capital compared with other provinces (Central Bank of Sri Lanka, 2024)”.* |
| Line 359 – provide a brief explanation of the respondent’s eligibility criteria | Thank you for your comment. In response, we have added the eligibility criteria used in the study, as presented in lines 401 - 406, as follows:  *“Eligible respondents were owners of locally owned SMEs operating in the Western Province of Sri Lanka who were directly involved in financial decision-making. The study included only firms with 11–300 employees, in line with the national SME definition, and excluded foreign-owned enterprises due to their systematically different performance characteristics. Participation was voluntary, and only respondents who provided informed consent were included”.* |
| Line 363- Data from Fforeign-owned SMEs | As you mentioned, we have corrected the grammatical error visible in line 400, as follows:  “foreign-owned SMEs” |
| Line 393 – “and more tailored toward CEOs and managers”, please rephrase this to “..tailored in the context of firm-level study. | Based on the valuable comment, the sentence in lines 434–437 has been revised as follows:,  *“This study adopted the scale developed and validated by* *Diéguez-Soto, Martínez-Romero (51),* *as it is more practical for the present research and tailored in the context of firm-level study”* |
| Instead of borrowing the scale, use an academic tone such as adopted/adapted etc | Based on the valuable comment, the entire document has been proofread by a professional, and the sentence in lines 434–437 has been revised as follows:  *“This study adopted the scale developed and validated by* *Diéguez-Soto, Martínez-Romero (51),* *as it is more practical for the present research and tailored in the context of firm-level study”* |
| Line 427 – “most businesses employed between 1 and 10 employees (49%)” – Please check this, as it contradicts the definition of small and medium-sized enterprises in Sri Lanka specified in Lines 362 & 363. | Thank you for pointing out the apparent contradiction. Upon review, we confirm that the SME definition used in this study is correct, with firms employing between 11 and 300 employees. The previously mentioned line regarding the number of employees was mistakenly linked to years of experience (49%) and has now been removed to avoid confusion. |
| Line 438 – The construct of Fintech adoption is operationalised as Intention to adopt not existing adopters, and supported by the statement in Line 403. However, the operationalisation of fintech adoption as intention is not aligned with the discussion of the LR, nor the problem statement in the Introduction. My argument is that intention to adopt cannot possibly link to the actual performance, as only usage can imply performance, as supported in the literature section. | We sincerely thank the reviewer for identifying the conceptual inconsistency between intention-based measurement and firm performance outcomes.  Following this observation, we carefully re-examined the fintech adoption construct. We agree that intention to adopt cannot directly explain organisational performance, as performance effects can only arise from actual usage of fintech services.  In our study, the measurement of FinTech adoption initially comprised three items. However, as highlighted by the reviewer, the third item (FA3) captured intention or recommendation rather than realised behaviour. Accordingly, we removed this future-oriented item from the construct. Following this revision, the FinTech adoption variable exclusively reflects actual usage behaviour that is, the realised adoption of financial technology within the firm thereby ensuring stronger conceptual alignment and construct validity.  Therefore, fintech adoption in the revised model represents:  Current utilisation of fintech services, and  Continued use of implemented fintech solutions,  which together operationalise actual adoption rather than behavioural intention.  We re-estimated both the measurement and structural models after removing the future-oriented item. The revised construct demonstrates improved conceptual alignment with the literature review and problem statement, as well as stronger theoretical justification for examining its impact on firm performance. Since the construct now captures realised usage, the linkage between fintech adoption and firm performance is theoretically and empirically defensible. |
| Line 479 – please use standard p-value reporting in Table format, as the latest APA format has different guidelines for Table and in-text reporting | Thank you for your comment. All p-values throughout the document have been revised according to the latest APA style. Previously reported as 'P value = 0.05', they are now presented as '.05' in tables and appropriately formatted within the text. |
| Researchers are required to provide results for bootstrapping analysis called Confidence Interval Lower Limit and Upper Limit based on the recommendation by Preacher and Hayes (2008) to add the robustness of the significance t-value result. An additional test is also necessary to specify the effect size of the mediation path. | Thank you for the valuable comment. Accordingly, we have added the Confidence Interval (Lower and Upper Limits) and effect size using bootstrap analysis as per Preacher and Hayes (2008) in lines 535–536, as follows:   |
| Line 567 – “Aligning with KBV theory and self-developed theories in literature” – This is confusing, as the self-developed theory has not been mentioned at all. In fact, researchers must consistently focus on firm-level theory, not individual-level theory. | We thank the reviewer for highlighting this issue. The reference to “self-developed theories” has been removed to avoid confusion. The theoretical framing has been revised to consistently anchor the study in the Knowledge-Based View (KBV), with financial literacy conceptualised as a firm-level knowledge resource rather than an individual-level construct.  Line 188 to 197  “*Drawing from these theoretical grounds, financial literacy has been framed as a strategic knowledge resource that demonstrates primary characteristics of the KBV. Financial Literacy incorporates experience-based knowledge and specialized knowledge regarding financial markets, how to allocate resources efficiently, assess investment performance, and to manage risk in the area/domain of finance. Financial Literacy is primarily tacit in nature, which means that it cannot be fully transferred or duplicated by formal education alone; rather, it is developed through experience and context-based application. Therefore, financially literate owner-managers develop knowledge-based assets that are hard for others to replicate or imitate, thus providing firms with potential sources of competitive advantage.”.*  *Line 242 to 256*  “*The KBV posits that firm performance is driven by the effective creation, integration, and application of knowledge-based resources. Consistent with KBV, the value of knowledge is context-dependent and does not generate strategic advantage in isolation. In the present study, financial literacy is conceptualized as a firm-level knowledge resource that enhances decision-making and operational effectiveness, rather than as an individual-level attribute. Accordingly, the contribution of financial literacy to business outcomes is contingent upon how such knowledge is embedded and utilized within organizational processes. Aligning with KBV and prior empirical evidence, this study proposes the following hypothesis*”. |
| Overall, this paper requires serious attention for language and structural editing. | Thank you for your recommendation regarding language correction. We have had the document reviewed by an official proofreader and made the necessary language corrections accordingly. |
| Check the overall format of citations. | All citations have been updated to comply with the journal’s guidelines. |
|  |  |

- **Reviewer Two**

| **Abstract** | |
| --- | --- |
| **Reviewers’ Comment** | **Response to the Comment** |
| 1. The judgment using a sample in the Western Province of Sri Lanka must be explained as a limitation. | The limitation related to the use of judgement sampling and the focus on the Western Province of Sri Lanka has now been explicitly acknowledged and discussed in the Limitations and Future Research section as follow Line 691 to 697.  *“In addition, the sample was selected using a judgement sampling technique and was confined to firms located in the Western Province of Sri Lanka. While this region represents a major economic and commercial hub, the use of a non-probability sample and a geographically restricted context may limit the generalizability of the findings to firms operating in other provinces or regions. Future research is therefore encouraged to employ probability sampling methods and extend data collection to other geographical areas to enhance the external validity of the results”.* |
| 2. The background has already presented the research urgency, namely, the lack of literature on financial literacy at the SME level; however, studies on financial literacy in developing economies already exist. What, then, constitutes the distinguishing factor of this study? | *Thank you for this important observation. While we acknowledge that studies on financial literacy in developing economies do exist, the distinguishing contribution of this study lies in its conceptual, contextual, and methodological integration, rather than in examining financial literacy in isolation.*  *First, this study explicitly conceptualizes financial literacy as a firm-level strategic knowledge resource, grounded in the Knowledge-Based View (KBV), rather than treating it as an individual behavioral trait. Much of the existing literature in developing economies focuses on individual or household-level outcomes (e.g., savings, borrowing, personal investment decisions). In contrast, our study advances the literature by theorizing and empirically validating owner-manager financial literacy as an organizational capability that influences firm performance, thereby extending financial literacy research into the domain of strategic management and SME performance.*  *Second, the study uniquely examines the combined and simultaneous mediating roles of access to finance and FinTech adoption in the financial literacy–firm performance relationship. Prior studies in developing economies tend to:*   - *examine financial literacy and firm performance directly,* - *or focus on access to finance or FinTech adoption independently,* - *or concentrate predominantly on developed-country contexts.*   *By integrating both traditional (access to finance) and digital (FinTech adoption) financial mechanisms within a single empirical framework, this study responds to fragmented findings in prior literature and provides a more holistic explanation of how financial literacy translates into superior firm performance in resource-constrained environments.*  *Third, the contextual setting of Sri Lanka provides an empirically underexplored yet theoretically relevant environment. Sri Lanka represents a developing economy characterized by:*   - *persistent macroeconomic instability,* - *underdeveloped capital markets,* - *heightened information asymmetry, and* - *increasing reliance on alternative digital financial solutions post-economic shocks.*   *These structural conditions intensify the relevance of financial literacy as a strategic capability and differentiate this study from prior research conducted in relatively stable developing economies or emerging markets.*  *Fourth, this study employs a firm-relevant financial literacy scale specifically designed for organizational decision-making, rather than relying on traditional individual-level literacy measures commonly used in prior studies. This improves construct validity and addresses a key methodological limitation highlighted in recent financial literacy–FinTech research, where insignificant or mixed results may stem from measurement misalignment with business contexts.*  *Finally, empirically, the study contributes by demonstrating partial mediation effects, showing that financial literacy influences firm performance not only directly but also indirectly through enhanced access to finance and increased FinTech adoption. This nuanced finding advances existing literature that often reports only direct effects or inconclusive relationships.*  *In summary, the distinguishing factor of this study is not merely its focus on financial literacy in a developing economy, but rather its theoretical framing, integrated mediation model, firm-level operationalization of financial literacy, and evidence from an underrepresented yet highly relevant national context. Together, these contributions meaningfully extend current knowledge on how financial literacy functions as a strategic enabler of SME performance in developing economies.* |
| 3. The Knowledge-Based View (KBV) is an appropriate framework for explaining financial literacy as a strategic knowledge resource. | Thank you for your insightful comment. We agree that the Knowledge-Based View (KBV) is an appropriate framework, and we have ensured its application to explain financial literacy as a strategic knowledge resource. |
| 4. Methodology Aligned with the Research Objectives PLS-SEM is suitable for complex models, moderate sample sizes, and the testing of mediating effects. | Thank you for your comment. We appreciate your feedback and note that this aspect has already been addressed in the study. |
| 5. The discussion section consistently and logically links the empirical findings with prior literature and the Knowledge-Based View (KBV) framework. However, the discussion remains relatively descriptive and has not yet fully explored potential macroeconomic factors and regulatory aspects. | The discussion section has been expanded to incorporate macroeconomic and regulatory considerations. Relevant literature has been cited to contextualise the findings within the Knowledge-Based View and the institutional environment faced by SMEs in developing economies. In line 613 to 625 as follow,  *“Beyond firm-level dynamics, SMEs in developing economies often operate under turbulent condi-tions of macroeconomic volatility, including inflationary pressures, fluctuating interest rates, credit tightening, and periodic economic shocks, which significantly influence financing and investment decisions [82, 122]. In such environments, the strategic value of knowledge, as emphasizsed by the Knowledge-Based View (KBV), becomes highly context-dependent [51, 123]. Financial literacy enhances firms’ ability to interpret macroeconomic signals, manage financial uncertainty, and adjust strategic decisions accordingly. Moreover, regulatory frameworks governing banking practices and FinTech adoption shape the extent to which financially literate firms can translate knowledge into performance outcomes. Stringent lending regulations and compliance requirements may restrict access to formal finance, while evolving FinTech regulations influence trust, perceived risk, and adoption decisions [121, 124]. Consequently, the positive effects of financial literacy on access to finance, FinTech adoption, and firm performance observed in this study are conditioned by prevailing macroeconomic and regulatory structures*”. |
| 6. The recommendations for SMEs and the government have clear practical and policy implications, particularly regarding the redesign of financial literacy programs and the promotion of FinTech. The study also acknowledges limitations related to the use of control variables, which are suggested as avenues for future research. | Thank you for your comment. We appreciate your feedback and note that the recommendations and limitations mentioned have already been addressed in the study. |
| 7. This article is ready for publication as it is relevant to the SME, finance, and managerial accounting literature. In addition, the article offers clear empirical contributions, although it still has reasonable methodological limitations typical of survey-based studies. | Thank you for your comment. We appreciate your positive feedback and note that the article has been prepared with attention to its empirical contributions and methodological considerations. |
